# The effects of the post-delay epochs on working memory error reduction

**DOI:** 10.1371/journal.pcbi.1013083

**Published:** 2025-05-13

**Authors:** Zeyuan Ye, Haoran Li, Liang Tian, Changsong Zhou

**Affiliations:** 1 Department of Physics, Hong Kong Baptist University, Hong Kong, China; 2 Centre for Nonlinear Studies and Beijing-Hong Kong-Singapore Joint Centre for Nonlinear and Complex Systems (Hong Kong), Hong Kong Baptist University, Hong Kong, China; 3 Institute of Transdisciplinary Studies, Hong Kong Baptist University, Hong KongChina; 4 Department of Physics, Washington University in St. Louis, St. Louis, Missouri, United States of America; 5 Institute of Computational and Theoretical Studies, Hong Kong Baptist University, Hong Kong, China; 6 Institute of Systems Medicine and Health Sciences, Hong Kong Baptist University, Hong Kong, China; 7 Life Science Imaging Centre, Hong Kong Baptist University, Hong Kong, China; UT Austin: The University of Texas at Austin, UNITED STATES OF AMERICA

## Abstract

Accurate retrieval of the maintained information is crucial for working memory. This process primarily occurs during post-delay epochs, when subjects receive cues and generate responses. However, the computational and neural mechanisms that underlie these post-delay epochs to support robust memory remain poorly understood. To address this, we trained recurrent neural networks (RNNs) on a color delayed-response task, where certain colors (referred to as common colors) were more frequently presented for memorization. We found that the trained RNNs reduced memory errors for common colors by decoding a broader range of neural states into these colors through the post-delay epochs. This decoding process was driven by convergent neural dynamics and a non-dynamic, biased readout process during the post-delay epochs. Our findings highlight the importance of post-delay epochs in working memory and suggest that neural systems adapt to environmental statistics by using multiple mechanisms across task epochs.

## Author summary

In daily life, we often need to store information temporarily (during a delay epoch) and retrieve it later when required. While this ability may seem simple, it poses significant challenges at the neural level. Neural activity is inherently highly variable (“noisy”), so how can we maintain accurate memory despite this variability? Previous research has primarily focused on neural processes during the delay epoch, however, the neural processes after delay (post-delay epochs) remain poorly studied. The post-delay epochs are critical for information retrieval—for instance, they are time periods when we write down something we just heard of, or, in a laboratory setting, when an animal makes a saccade to indicate a memorized color to earn a reward. In this study, we investigated the computational role of the post-delay epochs in helping noisy neural networks reduce memory errors. Our findings revealed that post-delay processes adapt to environmental statistics, and identified key neural mechanisms that reduce memory errors, highlighting the importance of post-delay epochs in supporting robust working memory.

## Introduction

Working memory is the ability to maintain information for a short period of time without external stimuli. It consists of a delay epoch for maintaining information, followed by post-delay epochs (for example, go and response epochs in typical delayed-response tasks [1,2]) for retrieving information [3]). Working memory tasks are fundamentally challenging because the neural system needs to maintain accurate information despite intrinsic stochastic noise. Without additional error-correcting mechanisms, the neural population activity will deviate from its original state, leading to large memory errors [4–7]. The neural mechanisms utilized by the neural system to mitigate these memory errors remain unclear.

Previous work has primarily focused on the neural dynamics during the delay [1,4,6–12]. For instance, the neural system can form attractors to represent some information values [1,8,9,13–15]. An attractor is a special neural population state; small deviations from the attractor state will be brought back. This unique property of attractors can stabilize neural population states against random deviations due to noise, thereby reducing memory errors. This attractor-based memory error correcting mechanism has been supported by experimental behavioral data [1], experimental neural recordings [16,17], and theoretical/simulation studies [6,9,18]. It can also be implemented by artificial neural networks using biologically plausible synaptic rules [13,19].

However, despite also being a key phase in working memory, the role of the post-delay epochs has largely been neglected. From the information processing perspective, the post-delay epochs act as a decoding process which maps the maintained neural state at the end of the delay to an output action [20–23]. Alterations to this mapping can result in significant changes in behavioral performance. The importance of decoding mapping can be demonstrated in the Brain-Computer Interface (BCI) experiments [23,24], where the BCI functions as a decoder, mapping neural population states to external actions (e.g., cursor movement). Alterations in the BCI’s decoding mapping would lead to significant action errors, necessitating relearning of the new decoding pattern by the animal [23,24]. Similarly, in working memory, the neural population state at the end of the delay must progress through the post-delay epochs, be decoded into muscle signals for an output action (e.g., a saccade). What is the decoding mapping from an end-of-delay neural state to output information? How does such mapping help reduce the memory error? What neural mechanisms during the post-delay epochs underpin this mapping? Addressing these questions is important for understanding the diverse processes, beyond just the neural dynamics during the delay epoch, of the neural system to reduce memory error.

In this paper, we trained artificial recurrent neural networks (RNNs) to perform a color delayed-response task, where the color in each trial was sampled from a prior distribution with a few high-probability colors (common colors) [9,25–27]. We found that the trained RNNs exhibited smaller memory errors on common colors, which aligns with previous behavioral experiments [1]. We found two main mechanisms that the RNNs used to reduce memory error. First, the neural system created attractors to encode common colors during the delay epoch (consistent with previous works [1,9]). Second, during the post-delay epochs, a large part of the neural population states was decoded to common colors, increasing the noise tolerance of the common colors. This noise-tolerant decoding mapping can be further understood by (1) attractor-based dynamics, and (2) a non-dynamic biased readout from the recurrent neural state to outputs during the post-delay epochs. Further, we used an approximate formula to naturally decompose the memory error into delay dynamic and decoding components and showed that neglecting the decoding component will lead to a failure in explaining the RNN’s memory error. Our results emphasize the importance of the post-delay epochs and propose experimentally testable predictions.

## Results

### RNNs trained in a biased environment exhibit human-like behaviour in the color delayed-response task

**Fig 1:**
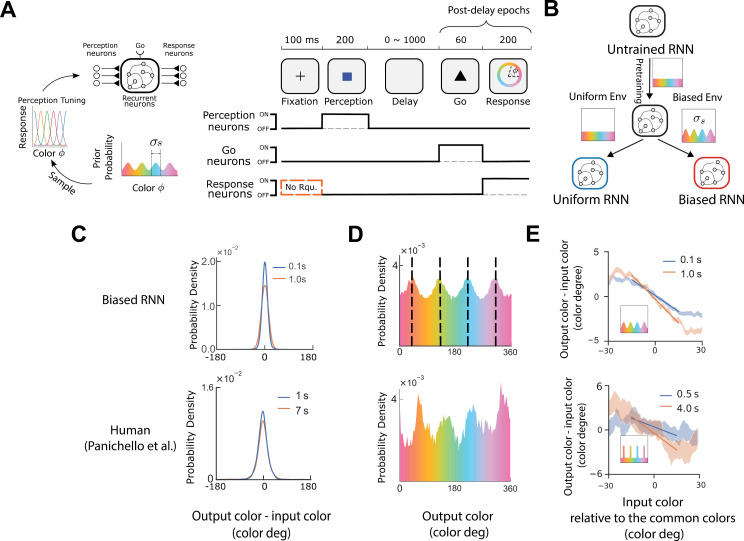
RNNs trained on a working memory task exhibit human-like behavior. (A) RNN architecture and the delayed-response task. An environment is defined by the prior probability function for the input color. In each trial, a color is sampled from the prior (four common colors = $40^{\circ }$, $130^{\circ }$, $220^{\circ }$, $310^{\circ };$ prior bump width is controlled by $\sigma_{s}$), and then sensed by the perception neurons. The RNN must maintain the sensed information during the delay epoch (delay length is randomly sampled from 0 to 1,000 ms), and then retrieve it in the response epoch. Except regularization terms, the loss function is applied only to the response neurons. These neurons have no requirements during the fixation epoch (No Rqu.) but should remain inactive during the perception, delay and go epochs. (B) Each RNN was trained progressively across multiple trials (see texts and Methods). Depending on the retrained environment prior, the final trained RNN was categorized as either a Uniform RNN (uniform prior) or a Biased RNN (biased prior). (C) The distribution of error (i.e., output color – input color), of the trained Biased RNNs ($\sigma_{s}=25^{\circ }$, n= 50) and human [1]. We performed 1,000 trials to each Biased RNN, with input colors uniformly sampled, and a delay length of either 100 ms or 1,000 ms (see other delay lengths in S1 Fig). Errors across 50 Biased RNNs were concatenated and normalized to draw this probability density. Bottom: Human data. (D) Upper: Same as panel (C), but directly showing the distribution of output colors (delay = 1,000 ms). Black dashed lines indicate four common colors in the training prior. Bottom: Human data. Note that humans had not yet been trained on biased prior color distribution. The bias observed here may arise from the biased color prior in the natural environment [1]. (E) Upper: We performed 1,000 trials on each Biased RNN (n = 50) using different input colors, and computed the trial errors as (output – input colors) which can be positive or negative. The error band is the standard error across trials and RNNs. RNN group’s trial error does not show statistical difference from zero when the input is a common color (S2 Fig). The solid line indicates the best-fitting line obtained through least-squares fitting, using data with x values (i.e., input color relative to the common colors) ranging from $-15^{\circ }$ to $15^{\circ }$. The inset shows the training environmental prior. Bottom: human participants were trained on a biased prior distribution (inset). Their performances during the last third of the training sessions are shown. Human data figures were plotted using the data published by Panichello et al [1].

We trained RNNs to perform a color delayed-response task [1]. The RNN consists of 12 perception neurons, 1 go neuron, 256 recurrent neurons, and 12 response neurons (Fig 1A and Methods). Each task trial consists of five epochs: fixation, perception, delay, go cue and response [1] (Fig 1A). The go epoch together with the response epoch act as a decoding process, transforming the neural state at the end of the delay into an output color. We refer to these epochs as the post-delay epochs (Fig 1A).

Specifically, In each trial, a color was sampled from an environmental prior distribution (a distribution with four peaks corresponding four frequently occurred common colors, analogous to the behavioral experiment [1]). This sampled color was then sensed by perception neurons according to the predefined von Mises tuning curves with shifting mean positions (Fig 1A). The elicited perception neural activities were then feedforwarded to recurrent neurons which maintained the color information during the delay epoch. The activation function for each recurrent neuron is tanh (see Methods). The delay duration was drawn from a uniform distribution U(0 ms, 1000 ms) to simulate the randomness in delay lengths in natural scenes.

After the delay, the go neuron received a cue signal initiating the response. The response neurons—equal in number to the perception neurons—were activated during the response epoch and were expected to reproduce the perception neurons’ earlier perception activity (see Methods). We averaged the activities of these response neurons over time and mapped them back into color information using a population vector method [6,28,29] (see Methods).

There are two sources of noise (see Methods): (1) Perception noise—a Gaussian noise added to perception neurons during the perception epoch, with standard deviation (std) denoted as $\sigma_{x}$; (2) Recurrent noise—a Gaussian noise added to the recurrent neurons throughout the trial except the fixation epoch, with std $\sigma_{rec}$. For simplicity, we set $\sigma_{x}=\sigma_{rec}=\;\sigma$, and referred σ as “noise level”.

We implemented a progressive training approach (Fig 1B and Methods) [30]. This approach involved two stages. First, in the pretraining stage, we trained randomly initialized RNNs in a uniform-prior environment. In the second stage, we retrained the RNNs in a new environment. We referred to the retrained RNNs as “Biased RNNs” if the new environmental prior was biased, and as “Uniform RNNs” if the new prior remained uniform. Both types of RNNs were trained with the same number of trials.

To evaluate the plausibility of using these RNNs in this study, we examined whether the trained Biased RNNs (n=50) exhibited behavioral patterns similar to those observed in previous human behavioral experiments [1,31] (see details in Methods). (1) We ran each Biased RNN with randomly sampled input colors (from a uniform distribution) under fixed delay lengths (see the results of 100 ms and 1,000 ms in Fig 1C, and for other various lengths in S1 Fig). The RNNs showed larger errors in longer delay (Figs 1C and S1). (2) When analyzing the output colors of the Biased RNNs (delay = 1,000 ms), we observed a distribution with four peaks near the four common colors—even though the input colors were uniformly sampled (Fig 1D). This suggests that the Biased RNNs developed an internal preference for common colors. (3) Further analysis of the input-output color relationship revealed a “neighborhood-attracting” effect: the output colors of the Biased RNNs tended to shift toward the common colors when the input colors were close to them (Fig 1E), with stronger “attracting” effect in longer delay (S1 Fig), consistent with previous findings [9,13,18,19]. These three results found in the Biased RNNs align with previous human behavioral studies [1].

### Biased RNNs show smaller memory errors than Uniform RNNs when the inputs are common colors

**Fig 2:**
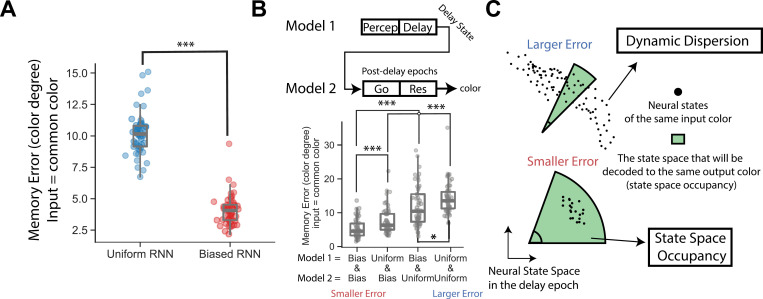
Post-delay epochs are crucial for memory error reduction. (A) Memory errors of trained Biased and Uniform RNNs with the input fixed at a common color ($40^{\circ }$). Each RNN was run for 5,000 trials with the input color set to a common color (delay = 800 ms). Outlier trials’ errors—defined as values beyond 1.5 times the interquartile range (IQR) above the third quartile or below the first quartile (or 1.5 times IQR for short)—were excluded. The memory error for each RNN was calculated as the root-mean-square error (RMSE) between the input and output colors across trials. Each dot represents the memory error of a single RNN. Fifty RNNs were tested for both the Biased and Uniform types. (***: $p<10^{-3}$, Wilcoxon rank-sum test, two tailed). (B) Cross-decoding demonstrates the critical role of the post-delay epochs in reducing memory error (i.e., comparing Bias & Bias vs. Bias & Uniform; Uniform & Bias vs. Uniform & Uniform). Upper panel: In a single cross-decoding trial, Model 1 was run with a common color input ($40^{\circ }$), and its neural state was collected at the end of the delay epoch (delay = 800 ms). This neural state was then transferred to Model 2 to initialize its state. Model 2 proceeded through the post-delay epochs and generated an output color. The memory error of a pair of Model 1–Model 2 was calculated by RMSE between the input and output colors across 500 trials. Fifty Model 1–Model 2 pairs were randomly sampled for each category combination (x-axis). Each dot represents the memory error of a single pair. ***: $p<10^{-3}$; *: $p\;<\;5\times 10^{-2}$; Wilcoxon rank-sum test, two tailed. Boxes indicate the interquartile range between the first and third quartiles with the central mark inside each box indicating the median. Whiskers extend to the lowest and highest values within 1.5 times the interquartile range. (C) A hypothesis breaking down the memory error reduction into two components: delay neural dynamics (dynamic dispersion) and the decoding mechanism (state space occupancy). Each axis represents one neural activity (recurrent neuron), and each dot indicates the neural state at the end of the delay for trials with a fixed input color (e.g., a common color). Memory error can be reduced when the neural states are more stable (i.e., smaller dynamic dispersion). The green region illustrates the portion of the state space that is decoded into an output color via the post-delay epochs. A larger state space occupancy of a specific color increases its tolerance to neural state dispersion, leading to smaller memory error for that color. In panel (A, B), the Biased RNNs were trained with $\sigma_{s}=12.5^{\circ }$.

The Biased RNNs’ preference for common colors suggests they may have smaller memory errors for these colors [1]. To test this, we ran the Biased RNNs for 5,000 trials with the input color fixed at a common color ($40^{\circ }$) and calculated the memory error as the root-mean-square error (RMSE) between the input and output colors across trials (memory errors for different input colors are shown in S3 Fig). Results showed that Biased RNNs had significantly smaller memory errors for a common color compared to Uniform RNNs (Fig 2A, $p<\;10^{-3}$, Wilcoxon rank-sum test, two-tailed). Although, the memory errors of common colors are not zeros, even after turning off the RNNs’ noises (S3B and S3C Fig). This might be due to the imperfection of the RNN training.

### Cross-decoding experiments reveal that the post-delay epochs are essential in memory error reduction

How do the Biased RNNs reduce the memory error for common colors? Post-delay epochs are crucial for retrieving maintained information at the end of the delay to output responses (e.g., output colors). However, the computational role of these post-delay epochs in working memory remain unclear.

To explore whether the post-delay epochs contribute to memory error reduction for common colors, we conducted a cross-decoding experiment. The cross-decoding experiment swaps the post-delay epochs of two RNNs, allowing isolating the effect of the post-delay epochs (Fig 2B, see details in Methods). Specifically, we randomly sampled two RNNs from either the Biased or Uniform category; the two models could belong to the same or different categories. Model 1 processed a trial with a fixed common color input. The neural states at the end of the delay were collected and transferred to Model 2’s recurrent neurons. Next, Model 2 directly ran through the post-delay epochs and output a color. The resulting memory error reflects the combined influence of Model 1’s pre-delay processing (including the delay) and Model 2’s post-delay decoding (excluding delay). We use a notation “Uniform & Biased” to indicate Model 1 was a Uniform RNN and Model 2 was a Biased RNN; similarly, “Biased & Biased” indicates both models were Biased RNNs.

A key step in the cross-decoding experiment is transferring Model 1’s neural representation to Model 2. We implemented two transfer methods (see Methods). The first method aligns individual neurons based on their preferred colors (see Methods). Neurons in each model were ranked by the color that elicited their highest activation, and neurons with the same rank in Model 1 and Model 2 were paired. In the transfer, the activation of each neuron in Model 1 was directly assigned to its corresponding neuron in Model 2. The second method applies an optimal population-level geometric transformation (see Methods). We ran 1,000 trials for both Model 1 and Model 2 with randomly sampled input colors and recorded their neural states at the end of the delay. We then fitted a geometric transformation—consisting of rotation/reflection, scaling, and translation in the neural state space—that minimized the distance between Model 1’s and Model 2’s representations. This fitted geometric transformation was used to transfer new, unseen neural states from Model 1 to Model 2 (see S4 Fig).

The results of both transfer methods are similar (Fig 2B for individual preferred color matching; S4 Fig for population-level matching). We found that the memory error of “Uniform & Biased” is smaller than that of “Uniform & Uniform,” and similarly, the memory error of “Biased & Biased” is smaller than that of “Biased & Uniform.” This indicates that the post-delay epochs play a key role in the memory error reduction.

### A hypothesis for explaining memory error reduction as a result of the combined effects of the delay epoch and post-delay epochs

The results of cross-decoding experiments highlight the role of the post-delay epochs in memory error reduction for common colors. What are the underlying neural mechanisms?

Previous research suggested that the neural system forms attractors to stabilize neural states during the delay, thereby reducing the memory error [1,8,9,13]. Building on this idea, we propose that the post-delay epochs further reduce memory errors by allocating large regions of the state space, for increasing the tolerance of outputs to random noises. Specifically, the post-delay epochs transform the neural state at the end of the delay into an output color. Geometrically, this process maps/decodes distinct regions of the state space (where axes are individual neural activities) to specific output colors. Colors occupying larger regions of the state space are more tolerant to noise, thereby reducing memory errors. For instance, even if neural states are noisy by the end of the delay, as long as they remain within the same region corresponding to a particular output color, the resulting output colors will remain stable and accurate.

In summary, we suggest that the memory error reduction of common colors arises from two key factors (Fig 2C): (1) decreased dynamic dispersion of neural population states during the delay (as suggested by previous studies [1,8]); and (2) the allocation of larger state space regions to common colors via the post-delay epochs. Note that the second factor only reflects the overall effect of the post-delay epochs. The underlying neural mechanisms involved within the post-delay epochs will be explored later in this paper.

### RNN’s neural activity is low-dimensional

**Fig 3:**
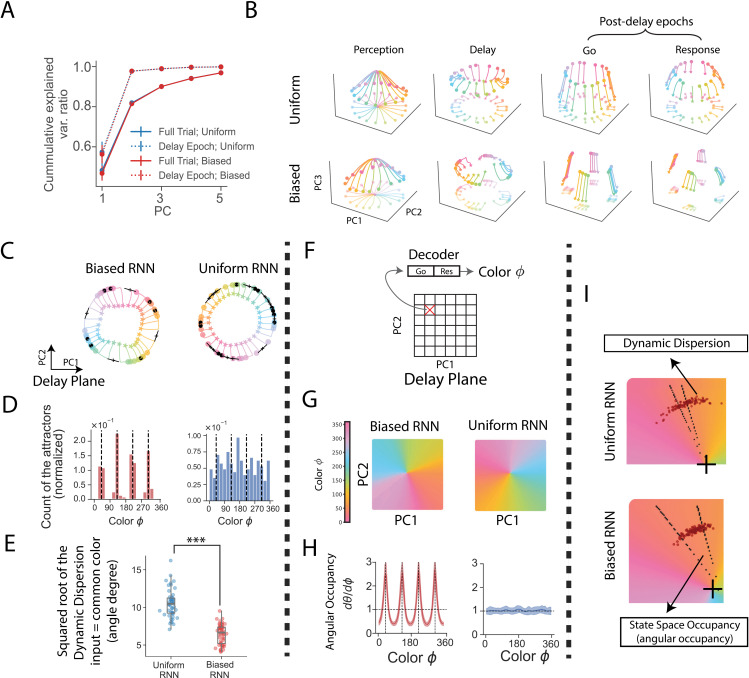
Reverse engineering of the RNNs revealed evidence for the hypothesized memory reduction mechanisms (smaller dynamic dispersion and larger state space occupancy). (A) Dimensionality of neural activity. Each RNN was run for 1,000 trials with randomly and uniformly sampled input colors. Neural activities were collected to compute cumulative explained variance ratio (var. ratio) using PCA. Dots and error bar represent mean and standard deviation (std) across 50 RNNs. Uniform: Uniform RNN; Biased: Biased RNN. Blue and red traces largely overlap. (B) PCA visualization of neural states. Stars denote the beginning of the epochs; dots represent the end. Each trajectory is one trial with color indicating the trial input color. Both 3D trajectories and their 2D projections were shown. (C) 2D visualization of two example RNN’s neural states during the delay epoch. This PC1-PC2 plane is also referred to as the delay plane. Black dots are the attractor points; crosses are saddle points whose long bars indicating the unstable (positive eigenvalue) directions. (D) Distribution of attractors. The trained Biased RNN are more likely to form attractors near common input colors (dashed lines). Results are based on concatenated attractors from 50 RNNs. (E) Reduced dynamic dispersion in Biased RNNs. To compute dynamic dispersion, we ran each RNN for 1,000 trials with the input color fixed at a common value (40°). Neural states at the end of the delay epoch (800 ms) were collected and projected onto the delay plane. Dynamic dispersion was defined as the variance of the angles of these neural states on the delay plane, after removing outlier trials (1.5 times IQR). Each dot represents the dynamic dispersion of a single RNN (n = 50 in total). ***: $p<10^{-3}$, Wilcoxon rank-sum test, two-tailed. Boxes indicate the interquartile range between the first and third quartiles with the central mark inside each box indicating the median. Whiskers extend to the lowest and highest values within 1.5 times the interquartile range. (F) Decoding refers to mapping neural states at the end of the delay to output colors. We sampled dense grid of points on the delay plane and decoded each point by running it directly through the post-delay epochs. (G) Decoding results of two example RNNs. Colors represent the decoded output colors. (H) We sampled a ring of points on the delay plane and decoded them by running through post-delay epochs. This establishes a relationship between angles (θ) on the plane and output colors (φ, i.e., decoded color). Angular occupancy, defined as dθ/dφ, measures the angular space occupied per unit of color. Solid lines indicate the mean, and shaded areas represent the std across 50 RNNs after excluding outlier RNNs (1.5 times IQR). (I) Joint effects of dynamic dispersion and angular occupancy in two example RNNs (see Methods). Each black dot represents the position of a neural state on the delay plane at the end of delay (800 ms) of a trial with the input color fixed at $40^{\circ }$. Dashed lines indicate the angular space enclosed by angles encoding $35^{\circ }$ and $45^{\circ }$ colors. Black crosses mark the center of the delay plane. In this figure, the Biased RNNs were trained with $\sigma_{s}=12.5^{\circ }$. To improve visualization, RNN noise was disabled ($\sigma_{x}=\sigma_{rec}=0$) after training, except in panel (E, I).

To test our hypothesis on memory error reduction, we analyzed the neural activities in the RNNs. We conducted multiple trials for each RNN (recurrent and perception noises were turned off), using uniformly sampled input colors. Recurrent neural activities were collected and projected onto the first few principal components (PCs) using Principal Component Analysis (PCA). The cumulative variance explained ratio suggests that the neural population activity is essentially low-dimensional (Fig 3A): requiring three dimensions to account for over 90% of the variance in neural activity across the entire trial, and only two dimensions when focusing exclusively on the delay epoch. This low dimensionality is also observed in RNNs designed with different number of perception/response neurons (e.g., see the results for RNNs with 6 and 20 perception/response neurons in S5 Fig), suggesting the internal representation dimensionality is robust to changes in the input/output dimensionalities (perception/response neurons).

This inherent low dimensionality of the neural activity allowed for direct visualization by projecting the activity onto the first three PCs, as shown in Fig 3B. During the perception epoch, neural states responded to input colors, moving in distinct directions. In the subsequent delay epoch, these states remained relatively stable, with minor lateral movements. Next, in the go epoch, the neural states shifted along the third principal component. Finally, in the response epoch, the neural states stabilized into a 2D ring-like structure. These visualizations illustrate the dynamic yet structured nature of neural activity during task execution.

### Biased RNNs are more likely to form attractors to represent common colors

With the intuition from visualization, next we tested the memory reduction hypothesis in Fig 2C. The first part of the memory error reduction hypothesis has been explored in previous studies [1,8,9,13]. Specifically, neural networks trained in biased environments tend to develop attractors near common colors [9,13]. These attractors stabilize neural states, thereby reducing memory errors [1,8,9,13]. The study of the delay epoch is not the focus of this work, but for completeness, we test whether this part of the hypothesis still holds true in our trained RNNs.

To develop an intuitive understanding, we analyzed two example RNNs from the Biased RNN and Uniform RNN categories, respectively. Neural trajectories during the delay epoch were projected onto their first two principal components, referred to as the “delay plane” (Fig 3C). Fixed points were identified by locating local minima in network speed (more precisely, these are slow points, which function similarly to fixed points [32], see Methods). Two types of fixed points were observed on the ring: (1) Attractors, which attract nearby states; (2) Saddle points, which repel nearby neural states along the direction of positive eigenvalues. Notably, in the Biased RNN, attractors predominantly clustered at four locations (Fig 3C). Additionally, these attractors exhibited more negative eigenvalues (indicating stronger attractive force) in more Biased RNNs (characterized by smaller prior $\sigma_{s}$, S6 Fig).

The four dominant locations may correspond to representations of the four common colors. To test this, we developed an RNN decoder (see Methods). This decoder is an exact replica of the original RNN. To decode a query neural state, the RNN decoder’s recurrent neural state is initialized as the query neural state. Without any delay, the decoder immediately progresses through the go and response epochs. The resulting output color is interpreted as the decoded color of the query neural state.

We decoded the attractors in both Uniform RNNs and Biased RNNs. The results revealed that in Biased RNNs, attractors were predominantly associated with common colors (Fig 3D). This tendency of attractors representing common colors is consistent with the result that the local minimum of memory error occurs at common colors (S3B and S3C Fig). Given the “attracting effect” of attractors, this finding implies that neural states may exhibit reduced dynamic dispersion when the input color is a common color. To test this, we directly measured dynamic dispersion (see Methods). Specifically, we ran multiple trials for each RNN with the input color fixed to a common color and the delay period fixed at 800 ms. Neural states at the end of the delay were collected, and their angular positions on the delay plane were calculated. The variance of these angles quantified the dynamic dispersion. The results (Fig 3E) revealed that dynamic dispersion for common colors was significantly lower in the Biased RNNs compared to the Uniform RNNs, supporting the hypothesis that the Biased RNNs reduce memory errors by reducing dynamic dispersion [1,8,9,13].

### Biased RNNs allocate larger angular occupancies to common colors

Next, we tested the second part of our hypothesis (Fig 2C): the Biased RNNs allocate larger state space occupancy to common colors. The overall effect of post-delay epochs is transforming neural states at the end of delay epoch to output colors. This process is identical to that of the RNN decoder (Fig 3F), which decodes a neural state by continuing its progression through the go and response epochs. To examine how color information is distributed across the state space, we applied the RNN decoder to a densely sampled grid on the delay plane (rather than using neural states from specific trials). Results from example RNNs indicated that color information is represented as angular positions on the delay plane (Fig 3G).

We investigated the quantitative relationship between angles and colors by sampling and decoding dense neural states along a ring on the delay plane (see Methods). This provided a numerical relation between the angle to the decoded color. The numerical derivative of angle with respect to color, referred to as angular occupancy, quantifies the amount of angular space allocated to a unit change in color. Results indicated that, in the Biased RNN, common colors exhibited larger angular occupancy (Fig 3H). In contrast, the Uniform RNNs displayed consistent angular occupancy across all colors. These findings suggest that the Biased RNNs reduce memory errors for common colors by dedicating more angular space to their representation.

To illustrate how neural dynamics and decoding jointly contribute to reducing memory errors, we analyzed two example RNNs (Fig 3I). Each RNN was run through multiple trials with a fixed common color input and a delay length of 800 ms. Neural states at the end of the delay were collected and visualized on the delay plane. Two dashed lines highlighted the region encoding colors from $35^{\circ }$ to $45^{\circ }$, centered around the common color $40^{\circ }$. Compared to the Uniform RNN, the Biased RNN exhibited both larger angular occupancy and smaller dynamic dispersion, supporting the proposed mechanism illustrated in Fig 2C.

### An approximate formula quantitatively links dynamic dispersion and angular occupancy to memory errors of common colors

Fig 3I provides a visualization of how the neural dynamic and decoding strategy jointly contribute to the reduction of memory errors for the common colors. To quantitatively capture their combined effects, we provided an approximate formula that relates dynamic dispersion and angular occupancy to the memory errors of common colors.

**Fig 4:**
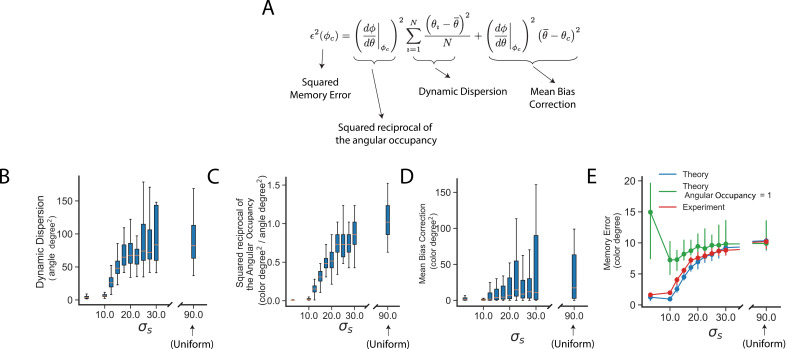
An approximate formula decomposes the memory error of a common color into a dynamic dispersion, angular occupancy, and a correction term. (A) An approximate formula for the squared memory error (see text and Methods). **(B, C,** D) Quantitative analysis of dynamic dispersion, the squared reciprocal of angular occupancy, and the mean bias correction for Biased RNNs trained under different biased environments (50 RNNs for each $\sigma_{s}$; see Methods for more detail). Smaller $\sigma_{s}$ indicates a narrower prior (see Fig 1A). Boxes indicate the interquartile range between the first and third quartiles cross 50 RNNs with the central mark inside each box indicating the median. Whiskers extend to the lowest and highest values within 1.5 times the interquartile range. (E) Comparison between the theoretical prediction (computed as the panel A using data from panel B, C, D) and the experimentally measured RNN memory error (see Methods). Dots represent the median memory error, and error bars indicate the first and the third quantiles across 50 RNNs for each $\sigma_{s}$. Experimental memory errors were computed over 5,000 trials per RNN with the input fixed to a common color (outlier trial errors were removed, 1.5 times IQR). For comparison, we also computed the theoretical prediction but assuming a trivial angular occupancy (equals to 1).


The central idea relies on Taylor expansion (see Methods). Color φ is a function of angle θ in the delay plane. Denoting the common color as $\varphi_{c}$, and its corresponding angle as $\theta_{c}$. Assuming that, when the trial input is the common color, the actual angle of neural state θ does not deviate significantly from the true color angle $\theta_{c}$. We expanded the color function as $\varphi (\theta )\approx \varphi_{c}+\left(d\varphi \text{/}d\theta {\;|}_{\varphi_{c}}\mathrm{\backslash rightleft}(\theta -\theta_{c}\right)$, where $d\varphi \text{/}d\theta {\;|}_{\varphi_{c}}$ is the reciprocal of angular occupancy. Inserting this approximation into the squared memory error $\in^{2}\left(\varphi_{c}\right)=\sum_{i=1}^{N}{\left(\varphi_{i}-\varphi_{c}\right)}^{2}\text{/}N$ (where $\varphi_{i}$ is the output color in trial i), we obtained an approximate formula (Equation 1 and Fig 4A) describing the memory error using angular occupancy ($d\theta \text{/}d\varphi {\;|}_{\varphi_{c}}$), dynamic dispersion ($\sum_{i=1}^{N}{\left(\theta_{i}-\overset{―}{\theta }\right)}^{2}/N$) and a mean bias correction ${\left(\overset{―}{\theta }-\theta_{c}\right)}^{2})$

$$\in^{2}\left(\varphi_{c}\right)={\left(\frac{d\varphi }{d\theta }{\;|}_{\varphi_{c}}\right)}^{2}\sum_{i=1}^{N}\frac{{\left(\theta_{i}-\overset{―}{\theta }\right)}^{2}}{N}+{\left(\frac{d\varphi }{d\theta }{\;|}_{\varphi_{c}}\right)}^{2}{\left(\overset{―}{\theta }-\theta_{c}\right)}^{2},$$ (1)

where the mean bias correction accounts for the mismatch of the trial averaged mean angle $\overset{―}{\theta }$ to $\theta_{c}$.

### Testing the approximate formula on RNNs trained on different environmental priors and different noise levels

We test the approximate formula (Equation 1) using RNNs trained under two scenarios: (1) varying the environmental prior while keeping the noise level fixed (Fig 4), and (2) varying the noise level while holding the environmental prior constant (S7 Fig). The environmental prior was modulated by the parameter $\sigma_{s}$, which controls the width of the prior distribution (Fig 1A). For simplicity, both the perceptual and recurrent noise were set to have equal standard deviations, collectively referred to as the noise level (see Methods).

For each trained RNN, we computed three key components: dynamic dispersion, the squared reciprocal of angular occupancy, and the mean bias correction (see Methods). These quantities were combined via Equation 1 to yield a theoretical prediction of memory error. To obtain experimental memory errors, we ran 5,000 trials per RNN using a fixed set of common colors as inputs and measured the RMSE between the input and output colors.

We found that decreasing $\sigma_{s}$—corresponding to a stronger prior over common colors—led to lower dynamic dispersion and higher angular occupancy (Fig 4B and 4C). Increasing the noise level resulted in higher angular occupancy (S7B and S7F Fig). Overall, the theoretical predictions closely matched the experimental memory errors (Figs 4E and S7H). To assess the contribution of angular occupancy, we recalculated the theoretical predictions but assuming a uniform angular occupancy (i.e., setting the term to 1 in Equation 1; Fig 4A). This modification led to poorer agreement with the experimental errors, especially for small $\sigma_{s}$ (Fig 4E) and high noise levels (S7H Fig). These results underscore the critical role of angular occupancy (and hence post-delay epochs) in accurately capturing memory errors in biased RNNs.

### Larger angular occupancy is due to both decoding dynamics and biased readout in the post-delay epochs

**Fig 5:**
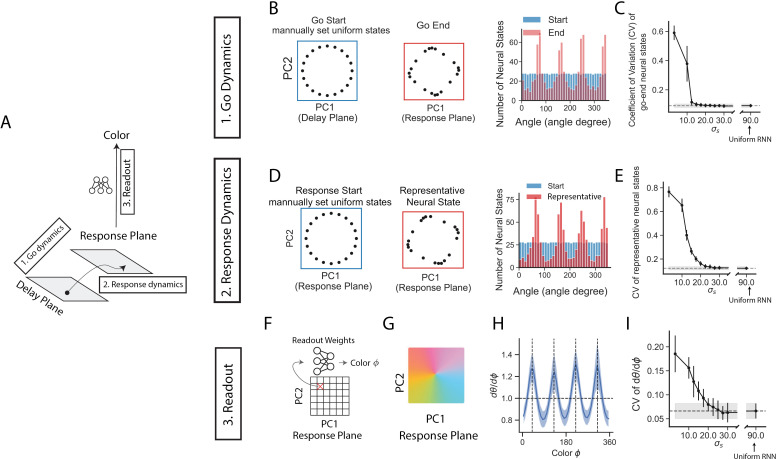
Larger angular occupancy (for common color) is due to both dynamics and biased readout mechanisms during the post-delay epochs. (A) The post-delay epochs roughly contain three stages (see Fig 3B): (1) Go cue drives neural states from delay plane to response plane; (2) Neural dynamics during the response epoch; (3) Readout from recurrent neural states to output color. (B) We isolated the effect of go dynamics, by firstly fixing neural states uniformly along a ring in the delay plane, then letting the neural states evolve through go epoch. The left two boxes show one example RNN’s neural states at the start and end of go epoch. Right: the distribution of 1,000 neural states of the same example RNN. (C) Coefficient of variation (CV) measures how non-uniform the neural states at the end of the go is, as computed by std divided by mean (see Methods). Dots and error bars are the mean and std of CVs across 50 RNNs (with outlier RNNs excluded, 1.5 times IQR). Dashed line and grey band indicate the mean and std of CVs for uniform RNNs ($\sigma_{s}=90^{\circ }$). (D, E) Similar to panels B and C, the neural states were initialized in the response plane and evolved throughout the response epoch. Representative neural states refer to the recurrent neural states temporally averaged over the entire response epoch. This averaging provides a more informative representation than neural states at the end of the response epoch, as the RNN’s output color is determined by these temporally averaged neural states (see Equation 7). (F) Dense mesh points on the response plane were directly mapped to output colors using a readout matrix and bias currents (see Methods, Equations 3 and 7), without any neural dynamics. (G) One example RNN’s decoded response planes. (H) Angular occupancy on the response plane. Line shows the mean of 50 RNNs ($\sigma_{s}=3^{\circ }$), and error band is the std, with outlier RNNs excluded (1.5 times IQR). Vertical dash lines: four common colors. (I) CV of angular occupancy, dots are the mean and error bars are std across 50 RNNs (outlier RNNs excluded, 1.5 times IQR), with horizontal dashed line and grey band highlighting the results of uniform RNNs ($\sigma_{s}=90^{\circ }$). The single example RNN shown in panel (B, D, G) was trained on prior $\sigma_{s}=3^{\circ }$.

We have shown that the angular occupancy is an important mechanism for reducing the memory error of the common colors. Angular occupancy indicates the end-to-end effect of the post-delay epochs. To investigate the underlying processes within the post-delay epochs that produce this non-uniform angular occupancy, we reverse-engineered the post-delay epochs using a divide-and-conquer strategy. The post-delay epochs can be roughly separated into three stages (Figs 3B and 5A). (1) Go dynamics: a go cue signal pushes the recurrent neural states from the delay plane to the response plane; (2) Response dynamics: recurrent neural states dynamically move during the response epoch (epoch length = 200 ms); (3) Readout: during the response epoch, recurrent neural states are readout (Equation 3 in Methods) into response neural activity, then mapped into output colors (Equation 7). The first two stages can be seen as continuing neural dynamics following the delay epoch (although there is a go cue input during the go epoch, altering the dynamic trajectory). They are also called decoding dynamics for short. The third stage, readout, does not involve dynamics. It simply multiplies the recurrent neural states by a readout matrix, adds a constant bias current, and transforms the results into output colors. One possible biological interpretation of the readout matrix might involve the neural connections from motor cortex to muscles (for actions), while the bias current might reflect the intrinsic property of each neuron (e.g., describing the diversity of neural firing threshold). We inspected possible mechanisms leading to non-uniform angular occupancy for each of these three stages (go dynamics, response dynamics, and readout).

To investigate the go dynamics, we sampled a uniform ring of neural states (Fig 5B) in the delay plane and allowed them to evolve through the go epoch (60 ms). At the end of the go epoch, we collected the evolved neural states and refitted their principal component (PC1–PC2) plane (response plane). We found that neural states accumulated to a few positions (Fig 5B). To quantify this accumulation, we computed the coefficient of variation (CV) of the neural-state distribution: larger CV indicates that neural states cluster more tightly around few positions. Fig 5C shows that neural states clustering becomes more pronounced at narrower environment prior $\sigma_{s}$. This biased motion during the go epoch might cause the non-uniform angular occupancy in the delay plane. For example, if all neural states converge to a single final position after the go epoch, they will all be read out as the same color, resulting in highly biased angular occupancy.

Similarly, biased motion during the response epoch may also contribute to non-uniform angular occupancy. As in the study of go dynamics, we sampled uniform neural states on a ring in the response plane and allowed them to evolve through the response epoch (200 ms). We then averaged these neural states over the response epoch duration, obtaining representative neural states (Fig 5D). Representative neural states provide a more accurate reflection of the network’s output than neural states measured only at the end of the response epoch (see Equation 7). We found that the representative neural states accumulate at a few locations, with more biased accumulation observed for a more biased environmental prior (small $\sigma_{s}$, Fig 5E). This biased dynamics during the response epoch may contribute to the increased angular occupancy. For example, if all neural states converge to a single location, all neural states would be decoded to the same output, resulting in highly biased mapping from neural states to output colors.

The above two dynamic mechanisms are reasonable. The go and response epochs themselves require a duration of time, necessitating the maintenance of information throughout the post-delay epochs, thus providing an opportunity for neural dynamics to play a role. However, it is important to recognize that the dynamics during the go and response epochs can be quite different from those of the delay epoch. The go pulses act as an input to the neural system, prompting a shift in neural states during the go epoch. This shifted space (response plane) may have different dynamic fields compared to the delay plane [33,34]. Therefore, it should not be regarded as merely a trivial extension of the delay dynamics.

Finally, we examined the non-dynamic readout process. We sampled a grid mesh of points on the response plane, and decoded them into output colors using the readout matrix along with the bias current (as elaborated in Equation 3, and Equation 7). The results (Fig 5H) revealed that the readout process inherently favors common colors by mapping larger angular regions to these colors. This bias becomes more pronounced with a smaller environmental prior $\sigma_{s}$ (Fig 5I), and with shorter response epoch (S8 Fig).

Together, these results suggest that biased motion during the go and response epochs, and the biased readout process are the possible reasons for larger angular occupancy for common colors in the delay plane.

### Go dynamics, response dynamics and readout processes each contribute to the memory error reduction

The biases identified in go dynamics (Fig 5C), response dynamics (Fig 5E), and readout (Fig 5I) imply that each of these three post-delay processes contributes to the reduction of memory errors for common colors. To test this, we simplified the post-delay epochs into a three-step model (S9 Fig). Given the low-dimensional nature of the RNNs (Fig 3B), we approximated neural states using their angles. At the end of the delay, neural states are characterized by their angles on the delay plane, which are then transformed through three steps: (1) go dynamics, resulting in new angles on the response plane; (2) response dynamics, further transforming the neural states into representative neural states with new angles; and (3) readout, where these representative neural states are converted into colors ranging from $0^{\circ }$ to $360^{\circ }$, which can also be interpreted as “angles.” Thus, each of these processes—go dynamics, response dynamics, and readout—can be conceptualized as nonlinear transformations between angles (S9 Fig).

To isolate the effect of each individual process on memory error, we initialized neural states on the delay plane to simulate the “scattered neural states” observed at the end of the delay. These states were then processed through the three post-delay steps, with only the targeted process being executed by the RNN, while the other two were replaced with uniform angle mappings. The final output color was compared to the input to evaluate the memory error.

We applied the above analysis procedure to both Biased RNNs (n=50) and Uniform RNNs (n=50). Compared to the Uniform RNNs, each process in the Biased RNNs resulted in smaller memory errors, supporting the idea that all three processes contribute to reducing memory error (S9 Fig).

## Discussion

Accurate retrieval of maintained information is critical in working memory. This retrieval process primarily happens during post-delay epochs, which map neural states at the end of the delay epoch to output responses (e.g., colors in this study). However, the computational role of post-delay epochs in memory errors remains unclear. In this study, we trained RNNs to perform delayed-response tasks (Fig 1) and demonstrated that post-delay epochs play a crucial role in reducing the memory errors of common colors (Fig 2B). Next, we reverse-engineered the trained RNNs and found that the post-delay epochs decode larger neural state spaces into common colors (Fig 3H and 3I)—a phenomenon important for quantitatively explaining memory errors (Figs 4E and S7H). This biased decoding was further explained by: (1) biased neural dynamics during the post-delay epochs (Fig 5B-E), and (2) a biased non-dynamic readout from neural activities to the output colors (Fig 5H-I). Overall, our analysis of the post-delay epochs provides a more complete understanding of the working memory process, complementing prior studies focused on the delay epoch [1,8,9,13].

In this study, we hypothesized that color memory error reduction is shaped by two key factors: (1) dynamic dispersion during the delay epoch and (2) the size of state space occupancy for a given color—an end-to-end effect of the post-delay epochs. To test this hypothesis, we analyzed two scenarios: RNNs trained with varying environmental priors (Fig 4) and different noise levels (S7 Fig). Beyond these two specific scenarios, we conceived that dynamic dispersion and state space occupancy are broadly important in contexts requiring high precision for specific colors. One potential example is reinforcement learning, where certain colors may be associated with higher rewards, even under a uniform environmental prior. In such cases, the neural system might adapt by reducing dynamic dispersion and/or expanding state space occupancy to minimize memory errors for high-reward colors. This prediction could be empirically tested through physiological experiments by assigning different reward values to specific information during animal training in delayed-response tasks [1,34–36], or explored in RNN models using reinforcement learning frameworks [37,38].

Concretely, this paper proposes several experimentally testable phenomena. First, when the input value prior probability is high, the dynamic dispersion (measured by the variance of repeated trials) should be smaller. Second, state space occupancy for high-probability values should be larger. The state space occupancy can be measured similarly to the numerical procedure described here, specifically, testing the animal on multiple trials with different input colors and collecting the neural population states at the end of the delay. These neural states, along with the animal’s output colors, provide a mapping from neural state space in the delay to an output color. Therefore, the output color to neural state differentiation could be estimated numerically, which is the state space occupancy. State space occupancies for high-probability colors should be larger. Finally, the approximate formula (Fig 4A) can also be tested. For example, train the animal to perform delayed-response tasks in different environmental priors [1,39], and then measure the dynamic dispersion, angular occupancy, and mean bias correction separately. Theoretical predictions can be compared with the animal’s experimental memory error.

In certain physiology experiments, the neural population state resides within a low-dimensional manifold and employs a ring-like structure to represent information [11,34,40], akin to the ring structure demonstrated in our trained RNNs. In such cases, the state space occupancy can be computed by determining the angle of the neural population state. However, we are aware that in many cases, neural manifolds exist in high dimensions, such as a highly curved trajectory within a high-dimensional space [5,7]. In these instances, we envision that state space occupancy does not rely on angular occupancy but rather possibly on “arc-length occupancy” [41]. Arc-length occupancy means the arc length used to represent a unit change of information. Measuring the arc-length occupancy of a highly curved trajectory can be challenging. Yet this challenge can be eased by the concepts and methods advanced in the recent geometric framework [42–45]. Various nonlinear dimensional reduction methods [45] (e.g., CEBRA [46], UMAP [47]) can project the manifold into a few latent dimensions for better visualization. Additionally, methods like SPUD [41], Gaussian process [48] or simple kernel smoothing [39] enable the direct parameterization of a high-dimensional curve by its arc length, facilitating the measurement of arc length occupancy for each information value. In the future, it would be interesting to explore the mechanisms of memory error reduction in high-dimensional and more complex manifold scenarios.

We modelled a classical color-delayed response task [9,11,13,18,19,49] that captures the most essential three stages of memory process: perception, maintenance (delay), and decoding (post-delay epochs). Beyond this classical experimental design, various memory task variants have been employed [1,34,39,50]. Some tasks, for instance, have no explicit go cue epoch [1,2]; in these cases, a color wheel may appear directly during the response epoch to prompt a response [1]. This constant visual feedback to the animal may lead to neural dynamics different from those shown in this paper. Additionally, some tasks involve multiple items displayed simultaneously for memorization [34,51], introducing further complexity. It remains unclear how our proposed framework—dynamic dispersion combined with state space occupancy—applies to these more complex tasks. In multi-item memory tasks, neural representations are unlikely to be restricted to an intrinsically one-dimensional manifold (as quantified by angular measures in this study). Instead, they might occupy higher-dimensional manifolds, such as surfaces with intrinsic two-dimensional structures [48,52]. This complexity raises several open questions: How to measure the state space occupancy of a higher-dimensional manifold? Does the neural system employed shared representations for correlated items [53], and disentangled geometry for statistically independent items [54,55]? Nevertheless, decoding is a ubiquitous process across diverse memory tasks, underscoring the need for further investigation of the decoding process (post-delay epochs) in complex tasks.

Beyond memory error reduction mechanisms, how RNNs adapt to the entire environmental prior is an interesting problem. Adaptation is not simply about reducing the memory error for all colors. As observed in S3B Fig, also in previous works [1,8,13], some low-prior colors exhibit larger memory errors. Thus, there is a trade-off for the RNN: how much error should be reduced for high-prior colors and how much should be increased for low-prior colors. This trade-off is unavoidable due to the neural mechanism. Introducing attractors to reduce memory error for common colors unavoidably leads to biased (or truncated) errors for nearby colors [1,8]. In the angular occupancy (Fig 3) part, enlarging common color’s angular occupancy will lead to smaller occupancy for other colors. How do RNNs balance memory errors among different colors remains unclear. Possible explanations can come from the Bayesian inference framework [56]. For example, Sohn et al. [39] assumes that the agent adapts to the environmental prior by making Bayesian inference in each trial. Environmental prior is the prior function, noise distribution is the likelihood function. Based on the prior and likelihood, they proposed that the agents need to output a memorized information value which equals to the mean of the posterior distribution. In this theory, while environmental prior is known, the effects of noise as a likelihood function remains unclear. Alternative theory suggests the network can be a generative model for reproducing the environmental prior [19]. RNN trained in this paper provides a ready-to-use models for theory pre-testing.

Biased RNNs exhibited reduced memory errors for common colors but increased errors for uncommon colors (S3B Fig). This inductive bias is advantageous when the testing environment closely resembles the training environment. However, it poses significant challenges when the testing environment differs substantially from the training environment. For example, if the common colors in the testing environment were uncommon during training, memory errors would be notably higher. This mismatch between training and testing conditions is known as distribution shift in machine learning [57]—a widespread issue in modern applications [57,58]. For example, in medical applications, models trained on data from a few hospitals are often deployed on data from entirely different hospitals [59]. In the context of this study, this issue is directly explained as a mismatch between the learned inductive biases in attractors/angular spaces and the testing data. The Biased RNNs presented in this study offer a toy model for preliminary investigation of inductive bias and out-of-distribution generalization.

## Materials and methods

### RNN architecture

The RNN consists of 12 perception input neurons, 1 go input neuron, 12 response output neurons and 256 fully connected recurrent neurons (Fig 1A, see similar activity patterns in networks with 6 or 20 perception/response neurons in S5 Fig). The dynamic equation of the RNN is [60]


$$x_{t}=(1-\alpha )x_{t-1}+\alpha \left(W^{rec}r_{t-1}^{rec}+W^{in}u_{t}+\sqrt{2\alpha^{-1}\sigma_{rec}^{2}}\in +b\right),$$ (2)

where $x_{t}$ is a 256-dimensional vector representing the activities/state of recurrent neurons (neural state) at time step *t*, and α = Δt / τ, where Δt is the time length for each compu*t*ational step, τ is the neuron membrane time constant. In this study, we set Δt=20 ms and α=1. Setting α= 1 has been used in previous studies, which simplifies the RNN into a discretized form that has been shown to be effective for training on working memory tasks [25,60]. The change of neural state $x_{t+1}$ is subject to 4 factors: (1) The inputs from the other recurrent neurons, $W^{rec}r_{t-1}^{rec}$, where $r_{t}^{rec}=\text{tanh}\left(x_{t}\right)$ and $W^{rec}$ is the recurrent weights but the diagonal elements fixed to 0 (self-connections are not allowed) [60]. (2) The inputs from perception and go neurons, $W^{in}u_{t}$, where $u_{t}$ consists of 12 components for the perception neurons and 1 component for the go neuron, and $W^{in}$ is the input weights. During the perception epoch, the perception neurons are entangled with an additive Gaussian noise with standard deviation $\sigma_{x}=0.2$ (except S7 Fig which varying $\sigma_{x}$). (3) The intrinsic recurrent noise in the RNN, $\sqrt{2\alpha^{-1}\sigma_{rec}^{2}}\in$, where ∈ is a 256-dimensional noisy vector drawn from the standard normal distribution at each time step, and $\sigma_{rec}\;=\;0.2$ (except S7 Fig, which varying $\sigma_{rec}$) is the recurrent neural intrinsic noise strength. For simplicity, we fixed $\sigma_{x}=\sigma_{rec}=\;\sigma$ where σ is the “noise level”. σ= 0.2 in all main figures except S7 Fig where σ was varied. (4) A constant bias current vector b.

The activities of the 12 response neurons are read from the $r_{t}^{rec}$,


$$z_{t}=W^{out}r_{t}^{rec}+b^{out}$$ (3)

### A task trial and RNN training

A trial contained a fixation, perception, delay, go, and response epochs. During the fixation epoch, the RNN does not receive any inputs (i.e., $u_{t}=0$). In the perception epoch, a color $\varphi_{s}$ is drawn from a prior distribution:


$$P_{s}\left(\varphi_{s}\right)=\frac{1}{4}\sum_{i}VM\left(\varphi_{s}-\mu_{i};\sigma_{s}^{2}\right),$$ (4)

where VM(·) is a von Mises function (the circular version of the normal distribution) with mean values at $\mu_{i}\in \text{\textbackslash{}\{}40^{\circ },130^{\circ },220^{\circ },310^{\circ }\text{\textbackslash{}}\}$:


$$VM\left(\varphi_{s}-\mu_{i};\sigma_{s}^{2}\right)=\frac{1}{2\pi I_{0}\left(1\text{/}\sigma_{s}^{2}\right)}\text{exp}\left[\text{cos}\left(\varphi_{s}-\mu_{i}\right)\text{/}\sigma_{s}^{2}\right],$$ (5)

where $\sigma_{s}$ is a free parameter to control the “width” of the prior distribution, and $I_{0}(\cdot )$ is the modified Bessel function of order 0 for normalization purpose. Units of $\varphi_{s}$, μ and $\sigma_{s}$ are converted from degree to rad before feeding into the von Mises function. A color is drawn from the above prior distribution and sensed by the 12 perception neurons. Perception neurons have tuning curves that are also von Mises functions $VM\left(\varphi_{s}-\mu_{p,\;i};{\sigma_{p}}^{2}\right)$ with $\mu_{p,\;i}=\frac{360^{\circ }}{12}i$ and $\sigma_{p}=\frac{1}{2}\frac{360^{\circ }}{12}$ for i=0, 1, …, 11 (Fig 1A). Therefore, during the perception epoch, the perception components of the input vector $u_{t}$ are $u_{t,i}=VM\left(\theta_{s}-\mu_{p,\;i};{\sigma_{p}}^{2}\right)+\in_{x}$ where $\in_{x}$ is a Gaussian noise with standard deviation $\sigma_{x}$, and the go component is 0 ($u_{t,12}=0$).

Next, during the delay epoch, all inputs are set to 0. The delay length is drawn from a uniform distribution *U* (0 ms, 1000 ms) for each trial. Then, in the go epoch, we set $u_{t,12}=1$ to indicate a go cue, while the rest of inputs and the outputs are 0. Finally, in the response epoch, the 12 response neurons are required to reproduce the same neural activity as the perception neurons during the perception epoch, i.e., ${\tilde{z}}_{t,\;i}=VM\left(\varphi_{s}-\mu_{p,\;i};{\sigma_{p}}^{2}\right)$ for i=0, 1, …, 11.

The loss function is


$$Loss=\frac{1}{T_{tot}}\sum_{t}m_{t}\left[\text{|}z_{t}-{\tilde{z}}_{t}{\text{|}}^{2}+\frac{1}{N_{rec}}\left(\beta T\text{|}W_{rec}{\text{|}}^{2}+\gamma \text{|}r_{rec}(x)\;+\;1{\text{|}}^{2}\right)\right],$$ (6)

where $m_{t}$ is a mask that equals to 0 in the fixation epoch and 1 in other epochs, $T_{tot}$ is the trial time length, $N_{rec}$ is the number of recurrent neurons, the regularization parameters β and γ constrain the recurrent weights and neural firing rates. The target output ${\tilde{z}}_{t}$ is 0 during the perception, delay and go epochs, and becomes ${\tilde{z}}_{t,\;i}=VM\left(\varphi_{s}-\mu_{p,\;i};{\sigma_{p}}^{2}\right)$ in the response epoch. We minimize this loss function via gradient descent using the Adam optimizer [61].

Directly training the RNN to optimize the above loss function is difficult, so we employed a progressive training protocol [30]: First, we trained the RNN with no noise, no regularization terms, uniform prior distribution, and a fixed delay epoch length of 0. Second, we retrained the RNN with a delay epoch length drawn from *U* (0 ms, 1000 ms). Third, we added noise and regularizations while retraining, producing a model we referred to as the pretrained RNN (Fig 1B). Finally, we retrained the pretrained RNN with a new prior distribution. If the new prior distribution remained uniform prior, we referred to the resulting model as the Uniform RNN (approximately corresponding to the prior $\sigma_{s}=90^{\circ }$); otherwise, we called it the Biased RNN.

### Mapping response neural activity into an output color

The actual response neural activity can be mapped back to a color using a population method. This mapped color is also referred to as the RNN’s output color. More precisely, we first average the response neural activity $z_{t}$ over the middle time window of the response epoch (from 60 ms to 140 ms, noting that the entire response epoch lasts 200 ms). Next, this averaged response neural activity $\overset{―}{z}$ is converted into an output color by a population vector method [6,28,29]:


$$\hat{\varphi }=\text{ang}\left(\sum_{m=1}^{N=12}{\overset{―}{z}}_{m}e^{i\mu_{p,\;m}}\right),$$ (7)

where ang(·) denotes taking the angle of a complex number, i here is the imaginary unit, and ${\overset{―}{z}}_{m}$ is the mth component of $\overset{―}{z}$, corresponding to the mth response neuron. We set $\mu_{p,\;m}=\frac{360^{\circ }}{12}m$ to align with the mean tuning of the perception neurons.

### Comparing trained Biased RNN behaviour with human behaviour

We investigated whether the trained Biased RNNs could reproduce key human behavioural features demonstrated in Panichello et al. [1]. We demonstrated key behaviour features in the bottom panels of Fig 1C, 1D and 1E using the publicly available data from Panichello et al. [1].

First, we examined whether the trained Biased RNN’s memory error increases with an increasing delay length. We ran each trained Biased RNN ($\sigma_{s}=25^{\circ }$,n=50) for 1,000 trials, using input colors randomly sampled from a uniform distribution, with the delay epoch length fixed at either 100 ms or 1,000 ms. Output-input color differences were computed. The resulting data across all RNNs and trials were concatenated to draw the distributions shown in Fig 1C.

Second, we examined whether the Biased RNN’s output color distribution remained uniform. We generated data the same as the above, but with the delay fixed at 1,000 ms. We concatenated output colors across all the Biased RNNs and trials. The resulting output color distribution is shown in Fig 1D.

Finally, we investigated whether the Biased RNNs developed an “attracting” effect around common colors. The data used here are the same as those described above, including both delay lengths of 100 ms and 1,000 ms. We shifted the input color by subtracting its nearest common color. The four common colors are $40^{\circ },130^{\circ },220^{\circ }$ and $310^{\circ }$. For example, the input color of $105^{\circ }$ was shifted to -$25^{\circ }$. Data from all the trials and Biased RNNs were concatenated. We plotted the output-input color difference against the shifted input color in the upper panel of Fig 1E, with the shaded area indicating the standard error across data. Additionally, we isolated data where the shifted input ranged from $-15^{\circ }$ to $15^{\circ }$, and fitted a straight line using an ordinary least squares regression, shown in Fig 1E.

### Cross-decoding experiment

Cross-decoding involves preparing delay neural states using one RNN model (Model 1) and decoding the prepared delay neural state using another RNN model (Model 2). Specifically, Model 1 was randomly chosen from either the Biased RNN or Uniform RNN categories. This model underwent 500 trials with a fixed input of a common color and a fixed delay length of 800 ms. Neural states at the end of delay were collected. Next, these end-of-delay neural states were used to set the recurrent neural states of Model 2 which was randomly selected from the second category (which may be the same as Model 1’s category). Model 2 processed these states over its post-delay epochs, output colors. Outlier trials were excluded (trials with output color – input color values 1.5 times the interquartile range above the third quartile or below the first quartile among the 500 trials). The RMSE across trials is the memory error of one Model 1-Model 2 pair. This procedure was repeated to gather memory error data for 50 randomly sampled pairs of each category combination, namely Bias & Bias, Bias & Uniform, Uniform & Bias, and Uniform & Uniform. The first category represents Model 1 while the second represents Model 2.

A key challenge in the above procedure is transferring the end-of-delay neural states from Model 1 to initialize the neural states in Model 2. In this paper, we used two approaches: (1) individual one-to-one neural matching, which involves comparing the preferred color of individual neurons (as detailed in *Methods: Transferring Model 1’s representation to Model 2’s representation via one-to-one neural matching in the cross-decoding experiment*); and (2) population matching, which identifies the geometric transformation that minimizes the distance between the two representations (as detailed in *Methods: Transferring Model 1’s representation to Model 2’s representation via population matching in the cross-decoding experiment*).

### Transferring Model 1’s representation to Model 2’s representation via one-to-one neural matching in the cross-decoding experiment

A traditional approach to determining a single neuron’s preferred color involves running the RNN with multiple input colors and identifying the input that elicits the largest neural response as the neuron’s preferred color. However, this method is problematic for the Biased RNNs, as the neural state shifts during the delay epoch to positions that no longer encode the original input color. To address this issue, we employed the following procedure to determine each neuron’s preferred color.

We ran the RNN for 1,000 trials with randomly sampled input colors and collected the neural states at the end of the delay period (800 ms). These neural states were used to fit a PC1-PC2 plane, referred to as the “delay plane” (Fig 3C). To ensure sufficient diversity in neural states, we computed the average distance of the neural states to their center and resampled dense neural states along a circle on the delay plane. This resampling step prevents the neural states from being overly concentrated near attractors (Fig 3C).

Next, we set the RNN’s recurrent neural states to the resampled states and, without introducing any further delay, allowed the RNN to directly process the go and response epochs, producing output colors. This process created a dataset mapping neural states to their corresponding output colors. Because a neural state is a vector of all individual neural activations, this also established a mapping between individual neural activations and their associated output colors. The output color that maximized a single neuron’s activation was then defined as its preferred color.

Having the above procedure estimating the preferred color of each neuron, we transfer Model 1’s neural states to Model 2’s neural states in the cross-decoding experiment as follows. We calculated the preferred colors of all recurrent neurons in Model 1 and ranked them from $0^{\circ }$ (smallest) to $360^{\circ }$ (largest). The same procedure was applied to Model 2. Neuron pairing was achieved by matching neurons with the same rank across models. This approach allowed the prepared delay neuron state in Model 1, consisting of 256 neurons, to map one-to-one with the recurrent neural state in Model 2, which also contains 256 neurons.

### Transferring Model 1’s representation to Model 2’s representation via population matching in the cross-decoding experiment

We developed a population matching method, i.e., rotation-translation-scaling (RTS) matching method (mostly based on Procrustes analysis [62]) to align two sets of observations, X and Y, each containing n sample points in $\begin{array}{c} {\mathbb{R}}^{d} \end{array}$ where d is the number of neurons. The goal is to find an orthogonal matrix $R\in {\mathbb{R}}^{d\times d}$ (representing a rotation/reflection), a uniform scaling factor s ∈ℝ, and a translation vector $\begin{array}{c} \text{\textbackslash{}textit\{t}\}\in {\mathbb{R}}^{d} \end{array}$ such that the transformed version of X best matches Y in a least-squares sense. Concretely, we seek to minimize the Frobenius norm of the difference between the transformed X and Y:


$$\min_{s,R,t}|\text{|}Y-(sRX+t){\text{|}|}_{F}^{2}.$$ (8)

We first subtracted the mean from each dataset. Let $\begin{array}{c} \mu_{\text{\textbackslash{}textit\{x}}\}=(\sum \text{\textbackslash{}textit\{x}{\}}_{i})\text{/}n \end{array}$ and $\begin{array}{c} \mu_{\text{\textbackslash{}textit\{y}}\}=(\sum y_{i})\text{/}n \end{array}$, where $x_{i}$ and $y_{i}$ are row vectors in ${\mathbb{R}}^{d}$. We then define the centered data $\begin{array}{c} X_{c}=X-\mu_{\text{\textbackslash{}textit\{x}}\} \end{array}$ and $\begin{array}{c} Y_{c}=Y-\mu_{\text{\textbackslash{}textit\{y}}\} \end{array}$.

We proceeded by forming a matrix $M=Y_{c}^{T}X_{c}$. This matrix captures the cross-covariance-like relationship between the centered observations. We then performed the singular value decomposition of M, namely $M=U\Sigma V^{T}$, where U and V are orthogonal matrices, and Σ is a diagonal matrix containing the singular values (implemented by numpy.linalg.svd in Python). From this factorization, we recovered the orthogonal transformation that best aligns $X_{c}$ to $Y_{c}$ by setting $R=UV^{T}$. The determinant of R may be either +1 (rotation only) or -1 (with reflection).

Once R is fixed, we determined the uniform scaling factor s by minimizing the sum of squared differences between $Y_{c}$ and sXRT. This leads to a closed-form solution for s:


$$s=\frac{\text{trace}(\Sigma )}{\sum_{i=1}^{n}\text{|}|x_{c,i}{\text{|}|}^{2}}$$ (9)

where $\begin{array}{c} \text{\textbackslash{}textit\{x}{\}}_{\text{\textbackslash{}textit\{c,i}}\} \end{array}$ is the i-th row in $X_{c}$.

The translation vector $\begin{array}{c} \text{\textbackslash{}textit\{t}\} \end{array}$ is finally determined by directly computing the residue between the mean of the transformed X to the mean of Y,


$$t=\mu_{y}-sR\mu_{x}$$ (10)

Knowing parameter R, s and $\begin{array}{c} \text{\textbackslash{}textit\{t}\} \end{array}$, we can transform new observations $\hat{X}$ to the representation of in Y space by $\begin{array}{c} \hat{Y}=s\hat{X}R+t \end{array}$.

We used this RTS matching method in the cross-decoding experiment as follows (S4 Fig).

The first step is to fit the parameters in the RTS matching method (R, s and t). We randomly sampled 1,000 colors, and used them as inputs to run 1,000 trials for both Model 1 and Model 2 (delay = 800 ms). Neural states at the end of the delay were collected, denoted as X and Y for Model 1 and Model 2, respectively. Next, R, s and $\begin{array}{c} \text{\textbackslash{}textit\{t}\} \end{array}$ parameters were computed as described above. This process finds the optimal rotation/reflection, scaling and translation transformation of X to Y, minimizing their Frobenius norm.

After fitting R, s and $\begin{array}{c} \text{\textbackslash{}textit\{t}\} \end{array}$, in the second step we fixed the input to a common color ($40^{\circ }$), and ran 500 trials on Model 1 (delay = 800 ms). Neural states at the end of the delay phase were collected and denoted as $\hat{X}$. These states, $\hat{X}$, were then transformed into $\hat{Y}$ via the fitted RTS matching. These transformed states, $\hat{Y}$, were then used to initialize the neural states of Model 2. Model 2 subsequently ran through the post-delay epochs to produce 500 output colors. After removing outlier trials, the memory error for a single Model 1-Model 2 pair was calculated as the RMSE across trials, represented as a single point in S4B Fig.

### Identifying the fixed points of the RNNs

The dynamic equation of the RNN (Equation 2) can be reformulated in the form of


$$x_{t}-x_{t-1}=F\left(x_{t-1}\right),$$ (11)

which defines the velocity field as v(x)=F(x\text/ Δt. In this study, we set Δt=20 ms. The global minimum points of speed v(x)=|v(x)|=0 are the fixed points. Specifically, we ran the trained RNN with 500 trials with stimuli equally distributed from $0^{\circ }$ to $360^{\circ }$. The neural states at the beginning of delay epochs were collected. According to the visualization in Fig 3, these neural states were likely near the ring on the delay. Hence these neural states were used as the initialization of searching point. Starting from these initial neural states, we searched for the global minima of speed using the gradient descent, where the independent variable is the position x. Due to the imperfection of the gradient descent algorithm, we might converge to local minima. Nevertheless, these local minimum points (i.e., slow points) are similar to the fixed points [32], hence they are also called fixed points in this paper. For each fixed point, we computed the eigenvalues and eigenvectors of its Jacobian matrix $J=\frac{\partial F\left(x\right)}{\partial x}.$ A fixed point is called an attractor if its largest eigenvalue is negative, while it is called saddle point if some eigenvalues are positive. The eigenvectors of the positive eigenvalues are projected and shown on the PC1-PC2 plane (Fig 3). Only fixed points near the delay ring were shown.

### RNN decoder: decoding neural states at the end of the delay to output colors

We built an RNN decoder to decode a delay neural state $x_{t}^{rec}$ to its corresponding color. This RNN decoder faithfully represents the post-delay epochs of the RNN. Specifically, an RNN decoder is the same as the original trained RNN, but its recurrent neural state is initialized as the to-be-decoded neural state. Then, the RNN decoder runs directly through the go and response epochs, and eventually produces an output color (Equation 7).

### Computing the dynamic dispersion of a fixed input color

Given an RNN, we computed the dynamic dispersion for a fixed common color input as follows. The first step is to fit the delay plane. We ran 1,000 trials with randomly input colors. Neural states at the end of the delay were collected for fitting a PC1-PC2 plane, namely the delay plane. In the second step, we ran 500 trials with the input color fixed to a common color (specifically, $40^{\circ }$ in Fig 3E). Neural states at the end of the delay were collected and projected into the previously fitted delay plane. We computed the angles of the projected neural states, denoted as $\theta_{i}$ where i indicates the i-th trial. Outlier angles (1.5 IQR below the first quantile or above the third quantile) were removed. Finally, dynamic dispersion is the variance of $\theta_{i}$. Each dot in Fig 3E represents the square root of the dynamic dispersion of one RNN.

### Decoding the delay plane of an RNN

We ran an RNN for 1,000 trials with randomly sampled input colors. The neural states at the end of the delay were collected and used for fitting the PC1-PC2 plane (the delay plane). We generated a mesh grid (50 × 50) on the delay plane and decoded these mesh points using the RNN decoder. The decoded output colors were shown in Fig 3G.

### Computing the angular occupancy of an RNN

We ran the RNN for 1,000 trials with randomly sampled input colors. The neural states at the end of the delay were collected and used for fitting the PC1-PC2 plane as the delay plane. In addition, we computed the average distance of the neural states to the neural states’ mean (i.e., center of these neural states). Using the average distance as the radius, we sampled a dense mesh of neural states along a circle on the delay plane and denoted each neural state’s angle as $\theta_{i}$. These neural states were then decoded by the RNN decoder to output the corresponding output colors. This procedure established a relation from angles (θ) to output colors (φ). Angular occupancy is defined as dθ/dφ, which measures the size of angular space occupied by a unit change of the color.

In Fig 3H, we estimated the angular occupancy of 50 RNNs, with solid line and error band representing mean and std of angular occupancy across RNNs (outlier RNNs, 1.5 times IQR, were removed).

### A demonstration showing the joint effects of dynamic dispersion and angular occupancy

In Fig 3I, we used an example to illustrate the effects of dynamic dispersion and angular occupancy. Here are the details.

We randomly selected one Biased RNN and one Uniform RNN. For each RNN, we conducted 1,000 trials with randomly sampled input colors (delay = 800 ms). The neural states at the end of the delay were collected for fitting the delay plane. We then created a grid of mesh points on the delay plane (50×50), and decoded them using the RNN decoder (see Methods). The decoded colors were shown as the background of Fig 3I (this procedure is identical to that described in Methods: decoding the delay plane of an RNN). In addition, the decoded colors were also used to indicate the region enclosed by $35^{\circ }$ to $45^{\circ }$. To identify one region boundary ($35^{\circ }$ or $45^{\circ }$), we located the top 30 points (among all the mesh points) whose decoded colors most closely matched the boundary color and marked these points in black in Fig 3I. We applied this method to find both region boundaries. Finally, we ran the RNN with 256 trials using a fixed input color of $40^{\circ }$ and collected the neural states at the end of the delay (delay length = 800 ms). These neural states were projected onto the previously fitted delay plane, visualized as grey dots in Fig 3I.

### A Taylor expansion approximation to the memory error

The squared memory error of a fixed common color $\varphi_{c}$ is


$$\in^{2}\left(\varphi_{c}\right)=\sum_{i=1}^{N}{\left(\varphi_{i}-\varphi_{c}\right)}^{2}\text{/}N,$$ (12)

where N is the number of trials, $\varphi_{i}$ is the RNN’s reported color in trial i. Approximately the RNN encodes a color by the angle (θ) of the delay plane, so the output color is a function of the neural state’s angle φ(θ). The angle encoding common color is denoted as $\theta_{c}$. Assuming during delay the actual neural state’s angle does not deviate from $\theta_{c}$ too much, then we can approximate φ(θ) using a Taylor expansion $\varphi (\theta )\approx \varphi_{c}+\frac{d\varphi }{d\theta }{\;|}_{\varphi_{c}}\left(\theta -\theta_{c}\right)$. Substituting this back to Equation 12 yields


$$\begin{array}{c} \begin{array}{c} \in^{2}\left(\varphi_{c}\right)=\sum_{i=1}^{N}{\left(\varphi_{i}-\varphi_{c}\right)}^{2}\text{/}N\approx {\left(\frac{d\varphi }{d\theta }{\;|}_{\varphi_{c}}\right)}^{2}\sum_{i=1}^{N}{\left(\theta_{i}-\theta_{c}\right)}^{2}\text{/}N={\left(\frac{d\varphi }{d\theta }{\;|}_{\varphi_{c}}\right)}^{2}\sum_{i=1}^{N}{\left[\left(\theta_{i}-\overset{―}{\theta }\right)+\left(\overset{―}{\theta }-\theta_{c}\right)\right]}^{2}\text{/}N\\ ={\left(\frac{d\varphi }{d\theta }{\;|}_{\varphi_{c}}\right)}^{2}\sum_{i=1}^{N}\frac{{\left(\theta_{i}-\overset{―}{\theta }\right)}^{2}}{N}+{\left(\frac{d\varphi }{d\theta }{\;|}_{\varphi_{c}}\right)}^{2}{\left(\overset{―}{\theta }-\theta_{c}\right)}^{2}, \end{array} \end{array}$$ (13)

where $d\varphi \text{/}d\theta {\;|}_{\varphi_{c}}$ is the reciprocal of the angular occupancy at common color, $\sum_{i=1}^{N}{\left(\theta_{i}-\overset{―}{\theta }\right)}^{2}\text{/}N$ is the dynamic dispersion, and the last term describes a correction from the actual neural state mean angle to the common color angle.

### Numerical procedure for testing the Taylor expansion approximation

To test this approximation formula (Equation 13), we computed the dynamic dispersion, angular occupancy and mean bias correction. To compute the dynamic dispersion of an RNN, we ran the RNN through 500 trials using a fixed common color input (delay = 800 ms). We collected neural states at the end of the delay and computed their corresponding angles $\theta_{i}$. After removing outlier angles (1.5 IQR below the first quantile or above the third quantile), we calculated dynamic dispersion as the variance $\theta_{i}$ (as described in Methods: Computing the dynamic dispersion of a fixed input color). The mean of $\theta_{i}$ was denoted as $\overset{―}{\theta }$. For angular occupancy, similar to Fig 3H, we sampled dense points along a ring in the delay plane. Each of these points was decoded by the RNN decoder, resulting in the angle-color mapping. Angular occupancy was then calculated as the numerical differentiation of angle with respect to color (detailed in Methods: Computing the angular occupancy of an RNN). Finally, for mean bias correction, we denoted $\theta_{c}$ as the angle of a sampled neural state (along the ring of the delay plane) whose output color most closely matched the common color. Knowing $\overset{―}{\theta }$ and $\theta_{c}$, we computed mean bias correction as written in Equation 13.

By knowing the dynamic dispersion, angular occupancy, and mean bias correction, we can predict the memory error through Equation (13), which is also referred to as the theoretical prediction in Fig 4E. To explore the importance of decoding (i.e., angular occupancy), as a comparison, we also computed the theoretical prediction but setting angular occupancy to 1.

To compute one RNN’s experimental memory error, we ran the RNN with 5,000 trials, fixing the input colors to the common color ($\varphi_{c}$), and collected the output colors denoted as $\varphi_{i}$. After removing outlier $\varphi_{i}$ (1.5 interquartile range below the first quantile or above the third quantile), the experimental memory error is the square root of the mean of ${\left(\varphi_{i}-\varphi_{c}\right)}^{2}$ (the squared root of Equation 12).

We tested Taylor expansion approximation under two scenarios. The first scenario is training RNNs under different environmental priors (varying $\sigma_{s}$, but keeping the noise level $\sigma_{x}=\sigma_{rec}=0.2$, see Equation 2). For each $\sigma_{s}$, we trained 50 RNNs, and estimated the dynamic dispersion, angular occupancy, mean bias correction and experimental memory error of each RNN as stated above. The second scenario is training RNNs with different noise levels (varying $\sigma_{x}=\sigma_{rec}=\sigma$, but keeping the environmental prior $\sigma_{s}=17.5^{\circ }$, see Equation 2). For each noise level σ, we trained 50 RNNs, and computed related quantities (dynamic dispersion etc.) as stated above. We used Python code scipy.stats.spearmanr to compute Spearman correlation along with its *p-*value.

### Biased neural state movement during the go and response epochs

We ran 1,000 trials of one example Biased RNN ($\sigma_{s}=3^{\circ }$) with randomly sampled input colors. We collected the neural states at the end of the delay period and used them to fit the delay plane. We then calculated the average distance from these neural states to their center (mean value). Using this average distance as the radius, we sampled 20 evenly spaced neural states in a ring on the delay plane. We used these 20 neural states as initial states and evolved them through the go epoch, collecting the final neural states. These end-of-the-go neural states were then used to fit a new PC1-PC2 plane (the response plane) and projected for visualization, as shown in the middle panel of Fig 5B.

To compute the neural state distribution for this example Biased RNN, we repeated the above procedure but with 1,000 neural states. We calculated the angles of both the initial neural states on the delay plane (which should show uniform distribution) and the final neural states on the response plane after the go epoch. These angular distributions are shown in the right panel of Fig 5B, demonstrating how the initially uniform distribution of neural states became biased following the go epoch.

The procedure for demonstrating biased movement in the response epoch follows a similar approach. We ran 1,000 trials using one example Biased RNN with randomly sampled input colors. We collected neural states from the beginning of the response epoch (which is also the end of the go epoch) and used these to fit the PC1-PC2 plane—the response plane. On this response plane, we sampled 20 example neural states in a ring and allowed them to evolve through the response epoch. We collected and averaged neural states across all time points during the response epoch. These time-averaged states, termed “representative neural states,” provide more meaningful information than end-of-response-epoch states since the RNN’s output color is based on the averaged response rather than the final response (see Equation 7). We projected these representative neural states onto the response plane for visualization, as shown in the middle panel of Fig 5D. Lastly, we repeated this procedure but with 1,000 neural states to obtain neural state distributions (right panel of Fig 5D).

### Decoding neural states on the response plane and computing the angular occupancy of readout

A neural state on the delay plane can be decoded into an output color by using the RNN decoder, which continuously runs the RNN through the go and response epochs. Similarly, a neural state in the response plane can also be decoded into an output color by continuously running the RNN. Since the response is already the last epoch, the “continuous running” only means reading the (recurrent) neural state out to the response neuron activity by using RNN’s readout weight $W^{out}$ and bias current $b^{out}$ (Equation 3). The response neuron activity is then transformed into an output color using the populational vector method (Equation 7). Note that no dynamics are involved since there’s no temporal component in this process. This decoding procedure is also called the Readout decoder. The properties of the Readout decoder are fully determined by the readout weights and bias currents.

To inspect the properties of the Readout process, we used the Readout decoder to decode mesh points on the response PC1-PC2 plane. Specifically, each RNN was run through 1,000 trials, neural states during the response epochs were collected for fitting the response plane (PC1-PC2 plane). After fitting, we created mesh grid points on the response plane. They were further decoded by the Readout decoder.

Similarly, we can also inspect the relation between angle and the color in the response plane. The procedure is analogous to what was described in the “Methods: Computing the angular occupancy of an RNN” section, except that the delay plane was replaced by the response plane, and Readout decoders were used instead of RNN decoders.

### Measuring the coefficient of variation (CVs) of neural states and angular occupancy

To measure the coefficient of variation (CV) of a function f(x), we discretized x into 36 bins, resulting in 36 discretized function values, denoted as $f_{i}$. The CV is defined as the std of the set $f_{i}$ divided by its mean. Note, the CV is a scale-free metric, remaining invariant under multiplication of f(x) by a constant.

To quantify the bias effect of go dynamics (right panel of Fig 5B), we obtained the distribution of neural states’ angles at the end of the go epoch for each RNN, as described in *Methods: Biased neural state movement during the go and response epochs.* This distribution serves as the function f(x) for computing the CV. Fig 5C presents the mean and standard deviation of CVs across 50 RNNs for each training environmental prior $\sigma_{s}$. The same procedure was applied to compute the CV of representative states and angular occupancy (Fig 5E and 5I).

### Measuring the effect of go dynamics, response dynamics and readout on the memory error of common colors

The CV describes the bias in neural dynamics or angular occupancy (Fig 5C, 5E, and 5I), but it does not directly measure the contribution of each process to memory error reduction for common colors. To quantify the effect of each process on memory error reduction, we used the following methods.

We simplified the post-delay epochs into three processes (see Figs 5A and S9): (1) go dynamics, (2) response dynamics, and (3) readout. Due to the low-dimensional nature of the trained RNN (Fig 3B), each process can be conceptualized as a nonlinear transformation of neural states, represented by their angles, across different planes. Specifically, go dynamics transform angles (θ) from the delay plane to the angles (α) on the response plane; response dynamics further transformed α into the new angles of representative neural states β on the response plane (temporal-averaged neural states since which were used for readout, see Equation 7 and main text); finally, readout transformed β into output colors φ, which could also be thought as “angles” ranging from $0^{\circ }$ to $360^{\circ }$.

To isolate the effect of a single process, we allowed only that process to run in the RNN, while manually setting the others as uniform transformations.

To set a process as uniform, we first established the initial conditions by identifying the angles corresponding to a common color ($130^{\circ }$). We ran the RNN 1,000 trials with random inputs and collected neural states at the end of the delay, at the end of the go epoch, and as representative neural states, as well as the corresponding output colors (φ). We computed these neural states’ angular values, thus establishing a chain of correspondences: θ~ α~ β~ φ.  Since we are primarily interested in the error for the common color, we retained only the angular values corresponding to the common color $\varphi_{c}$ (e.g., $130^{\circ }$) and denoted these angles with the subscript c. This chain of correspondences provided the initial conditions necessary for manually “ablating” a process into a uniform mapping. For example, to make go dynamics uniform, we replaced its transformation θ to α with $\alpha =\theta -\theta_{c}+\alpha_{c}$.

To compute the memory error when retaining only one non-uniform process (by the RNN), we initialized 500 evenly distributed angles on the delay plane, ranging from $\theta_{c}-\Delta$ to $\theta_{c}+\Delta$ where $\Delta =10^{\circ }$. These angles, denoted as $\theta_{i}$, simulate the “scattered” neural states corresponding to common color input.

To consider the effect with go dynamics only, we ran these initial neural states through the go epoch (with noise turned off) to obtain the resulting angles on the response plane $\alpha_{i}$. The subsequent processes were set to uniform transformations, without further using the RNN, ended up with output colors as $\varphi_{i}=\alpha_{i}-\alpha_{c}+\varphi_{c}$. The output colors were then used for computing the memory error, as RMSE over the set $\varphi_{i}$. Similarly, to consider the effect with readout process only, we generated the same set of initial neural states $\theta_{i}$. Next, without using RNN, we directly obtained $\beta_{i}=\theta_{i}-\theta_{c}+\beta_{c}$. Using $\beta_{i}$, we then inversed the response plane to get neural states finally decoded by the readout process of the RNN to obtain the output color $\varphi_{i}$, which then further used for computing the memory error. To consider the effect of response dynamics only, we computed $\alpha_{i}=\theta_{i}-\theta_{c}+\alpha_{c}$, then ran the RNN’s response epoch to obtain $\beta_{i}$, finally processing though a uniform readout process as $\varphi_{i}=\beta_{i}-\beta_{c}+\varphi_{c}$.

In S9 Fig, we showed the memory errors when only one process is non-uniform (run by the RNN), for 50 Biased RNNs ($\sigma_{s}=3^{\circ }$, same as the example used in Fig 5) and 50 Uniform RNNs.

## Supporting information

S1 FigMemory error and bias slope as a function of delay epoch duration (i.e., delay length).(**A**) For each RNN and delay length, we conducted 5,000 trials with randomly sampled input colors. The resulting output colors were used to compute the memory error, defined as the RMSE across trials, reflecting the curve width shown in Fig 1C. Dots and error bars represent the mean and standard error of the mean (SEM) across 50 RNNs. The correlation between memory error and delay length was computed using data concatenated from all RNNs and delay lengths. ***: $p\;<10^{-3}$, Pearson correlation test (computed via scipy.stats.pearsonr). (**B**) For each RNN and delay length, we conducted 5,000 trials with randomly sampled input colors. We then computed the slope of the relationship between (output − input) and the input color, using data using input colors within $\pm 15^{\circ }$ of the common color (similar to Fig 1E). Dots and error bars indicate the mean and SEM of slopes across 50 RNNs. Correlation between the slope and delay length was computed from data concatenating the slopes across RNNs and delay lengths. ***: $p\;<\;10^{-3}$, Pearson correlation test (computed via scipy.stats.pearsonr). All RNNs were trained under $\sigma_{s}=25^{\circ }$, which are the same RNNs as those used in Fig 1C–E.(TIF)

S2 FigBiased RNN output colors are centered around the common color when the input is a common color (e.g., $40^{\circ }$).(**A**) Same as Fig 1E but generated with a different random seed. (**B**) For each Biased RNN, we conducted 1,000 trials with the input color fixed at a common color ($40^{\circ }$). We averaged the trials errors (output – input) across all trials resulting in a single averaged trial error of one RNN, represented by a single dot in this panel. We repeated the same procedure for 50 RNNs, and two delay lengths (0.1s and 1.0s). Boxes represent the first and third quartiles, with the central line indicating the median. Whiskers extend to the minimum and maximum values within 1.5 times the interquartile range. There’s no statistically significant difference between the group medians and zero (p>0.5, Wilcoxon signed-rank test, two-sided), in both delay of 0.1 s and 1.0 s.(TIF)

S3 FigMemory error across different input colors for RNNs.(**A**) Memory errors of trained Biased ($\sigma_{s}=12.5^{\circ }$) and Uniform RNNs in a biased environment ($\sigma_{s}=12.5^{\circ }$). Each RNN was run for 5,000 trials with input colors sampled from a biased environment ($\sigma_{s}=12.5^{\circ }$). Errors from outlier trials, defined as values exceeding 1.5 times the interquartile range above the third quartile or below the first quartile, were excluded. The memory error of each RNN was calculated as the RMSE across trials. Each dot represents one RNN, with 50 RNNs used for each type. (***: $p<10^{-3}$, Wilcoxon rank-sum test, two tailed). (**B**) Top: The prior distribution of colors centered around a common color ($130^{\circ }$, black dashed line). Bottom: Memory error for various input colors. We ran 5,000 trials for each RNN (50 RNNs for each type) and each input color. Errors from outlier trials among the 5,000 trials were removed. The memory error of one RNN and one input color was computed as the square root of the average squared difference between the output and input colors across trials. The colored lines represent the mean memory errors across the 50 RNNs (after removing memory errors from outlier RNNs), with error bands indicating standard errors. (**C**) Same as the bottom of panel (B), but for Biased RNNs with all noise turned off.(TIF)

S4 FigCross-decoding with a population-level matching (see Methods).(**A**) Details of the method for performing cross-decoding on one pair of Model 1–Model 2 using rotation-translation-scaling (RTS) matching. In step one, 2,000 input colors were randomly sampled. Model 1 and Model 2 were run for 1,000 trials each, with neural states at the end of the delay collected and denoted as X and Y respectively. RTS matching finds the optimal scaling (s), rotation/reflection (R) and translation (t) to transform X by minimizing the Frobenius distance between the transformed X and Y. In step two, 500 trials were performed with input colors fixed to the common color. Model 1’s neural states at the end of delay ($\hat{X}$) were collected and transformed into Model 2’s representation ($\hat{Y}$). Model 2 ran through the post-delay epochs and finally generated output colors. (**B**) Same as Fig 2B in main text, but using RTS matching.(TIF)

S5 Fig RNNs trained with six (upper) and twenty perception/response units (bottom) exhibit low-dimensional representations. Figure illustrations are the same as those in Fig 3A and 3B.
(TIF)

S6 FigEigenvalues of the fixed points.(**Left**) We trained 50 RNNs for each prior $\sigma_{s}$. For each trained RNN, we identified its attractors and calculated the largest eigenvalue for each attractor. These largest eigenvalues were then averaged to represent the “attracting strength” of the RNN. The dots and error bars represent the mean and standard deviation of the averaged largest eigenvalues across the 50 RNNs. (**Right**) Same as the left, but applied to the largest eigenvalues of the saddle points. These results indicate that RNNs trained with narrower priors (smaller $\sigma_{s}$) tend to form stronger fixed points, which may explain the neural clustering observed in Fig 3C and 3E.(TIF)

S7 FigThe results for different noise levels also support that angular occupancy is an important factor to reduce memory errors at the common colors.(**A**) The delay plane for two example RNNs trained on low-noise (noise strength ${\sigma_{x}=\sigma }_{rec}=0.1$) and high-noise ($\sigma_{x}=\sigma_{rec}=0.3$). Colors indicate the decoded colors (i.e., output colors) by continuing the neural states on the delay through the post-delay epochs. (**B**) Angular occupancy. Dashed vertical lines are common colors. Solid blue/red lines indicate the mean of 50 RNNs. The error bands indicate std. (**C**) Neural dynamics during delay. The color of each trajectory indicates the input color of a trial. Stars are the beginning of delay; dots are the ends. Black dots are attractors, black crosses are saddles with long bar indicating the positive eigenvalue directions. (**D**) Number of attractors concatenated from 50 RNNs (normalized) for different colors. Dashed lines: common colors. (**E**) Same as Fig 4B. Increasing noise initially increase dynamic dispersion (noise from 0.10 to 0.22, Spearman correlation = 0.69, ${p<10}^{-3}$, two-tailed Spearman correlation test implemented by scipy.stats.spearmanr), but then decrease the dynamic dispersion (noise from 0.22 to 0.30, Spearman correlation = -0.33, ${p<10}^{-3}$, two-tailed Spearman correlation test implemented by scipy.stats.spearmanr). The counter-intuitive of decreasing dynamic dispersion might be due to an over-powerful attracting mechanism to counteract the effect of noise. For example, consider an extreme scenario where the noise is very high, input color information is destroyed completely during the trials. In this case, the RNN’s optimal strategy would be to output common colors for any inputs, as common colors have high priors. Therefore, the RNN might develop very strong attracting mechanisms under high noise conditions such that every trial the neural states would be quickly attracted to attractors to output common colors. (**F, G, H**) Same as Fig 4C, 4D, and 4E, but varying noise level. Fifty RNNs were trained at each noise level. (**I**) Experimental error same as (H), but input colors were randomly sampled from an environmental prior $\sigma_{s}=17.5^{\circ }$ instead of fixing to a common color. In this figure, all RNNs were trained on environmental prior ${\sigma_{s}\;=\;17.5}^{\circ }$. In panels (A, B, C, D, F), noise was turned off after training for better visualization.(TIF)

S8 FigRNNs trained on a shorter response epoch have weaker response dynamic bias and stronger readout bias.Training prior $\sigma_{s}=3^{\circ }$. The computational details are identical to those in Fig 5, except that the RNN models were replaced with those trained using long/short response (res.) epoch. (**A**) Long/short response epoch: an example RNN trained with a long (200 ms) or short (40 ms) response epoch length. Compared to the RNN trained with a long response epoch, RNN trained with a short response epoch shows smaller changes in neural states during the response epoch. (**B**) RNNs trained on long response epochs exhibit smaller bias as measured by the coefficient of variation (CVs) of representative neural states distributions (see Methods). Each dot represents one RNN’s representative neural state CV (red histogram in panel A). Boxes indicate the first, median and third quantiles across 50 RNNs. ***: $p<10^{-3}$, Wilcoxon rank-sum test. (**C**) RNNs trained on short response epoch have more biased readout. The solid line represents the mean of 50 RNNs, and the shaded error band indicates the standard deviation. Dash lines: four common colors. Outlier RNNs were removed (outside of 1.5 IQR). (**D**) Same as panel (B), but measuring the CV of angular occupancies.(TIF)

S9 FigThe effects of go dynamics, response dynamics and readout on memory error.**(A)** The post-delay process was divided into three distinct processes: (1) neural states at the end of the delay evolve through go dynamics, (2) followed by response dynamics, and finally, (3) are read out into colors. Due to the low-dimensional structure of the trained RNN (Fig 3A), each process can be simplified as a non-linear transformation of angles. For instance, the effect of go dynamics can be represented as a transformation of angles on the delay plane into new angles on the response plane. The angles at each stage are denoted as θ (end of the delay = beginning of the post-delay epochs), α, β and φ (color, also ranges from $0^{\circ }$ to $360^{\circ }$), respectively. The angle corresponding to a common color is indicated with the subscript c. To isolate the effect of a single process on the memory of the common color, we allowed only that process to run in the RNN while manually keeping the other two processes uniform (see Methods). (**B**) The memory errors associated with each of the three processes in Biased RNNs (n = 50, training prior $\sigma_{s}=3^{\circ },$ the same as the example RNN demonstrated in Fig 5B, 5D, and 5F–H) are smaller than those observed in Uniform RNNs. This suggests that each of the three processes—go dynamics, response dynamics, and readout—plays a role in reducing memory error. ***: $p<10^{-3}$, one-sided, Wilcoxon rank-sum test. Each dot is the result of one RNN. Outliers were omitted for visual clarity but were included in the statistical analysis. (**C**) Same as the panel B, but includes the visually omitted outlier RNNs in the panel B.(TIF)
