# Supplementary figures and images for: The effects of the post-delay epochs on working memory error reduction
Source: PLoS Comput Biol. 2025 May 13;21(5):e1013083. doi: 10.1371/journal.pcbi.1013083 (PMC12136468; doi:10.1371/journal.pcbi.1013083)

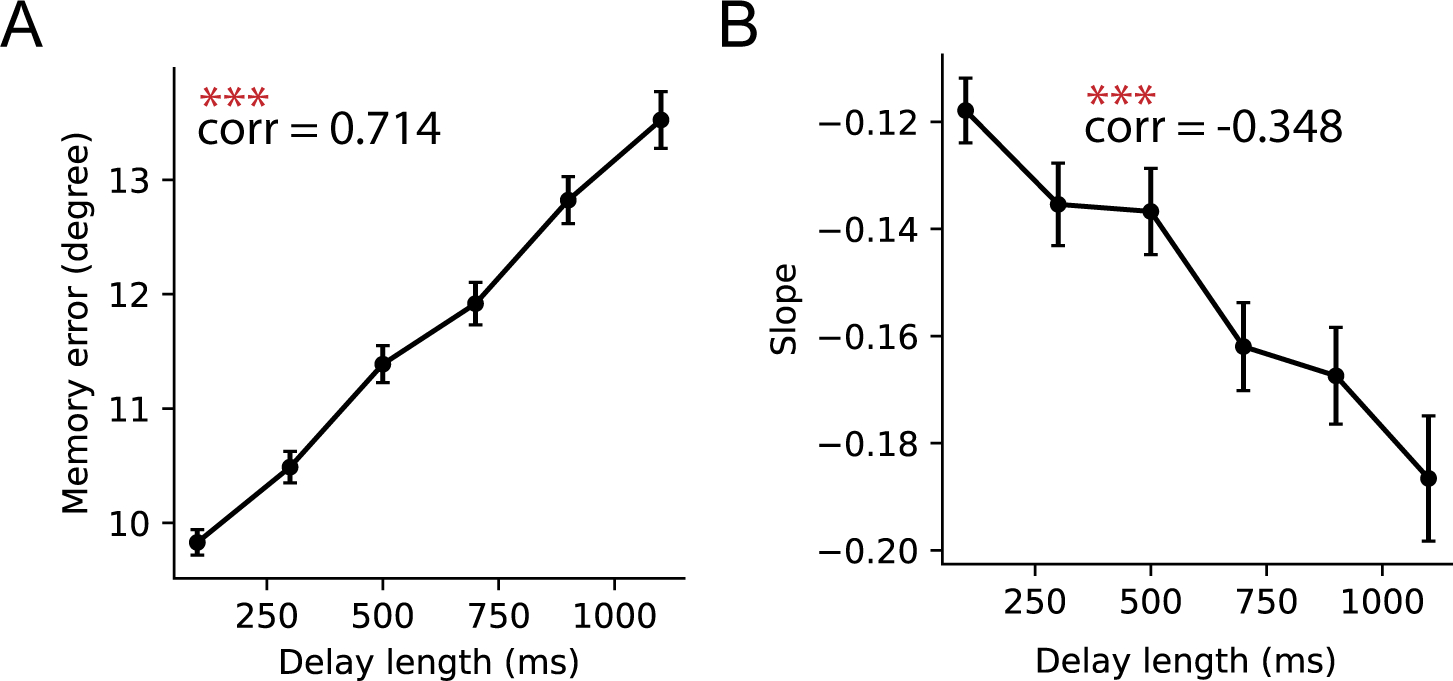

Supplement: S1 Fig — (A) For each RNN and delay length, we conducted 5,000 trials with randomly sampled input colors. The resulting output colors were used to compute the memory error, defined as the RMSE across trials, reflecting the curve width shown in Fig 1C. Dots and error bars represent the mean and standard error of the mean (SEM) across 50 RNNs. The correlation between memory error and delay length was computed using data concatenated from all RNNs and delay lengths. ***: p <10−3, Pearson correlation test (computed via scipy.stats.pearsonr). (B) For each RNN and delay length, we conducted 5,000 trials with randomly sampled input colors. We then computed the slope of the relationship between (output − input) and the input color, using data using input colors within ±15∘ of the common color (similar to Fig 1E). Dots and error bars indicate the mean and SEM of slopes across 50 RNNs. Correlation between the slope and delay length was computed from data concatenating the slopes across RNNs and delay lengths. ***: p < 10−3, Pearson correlation test (computed via scipy.stats.pearsonr). All RNNs were trained under σs=25∘, which are the same RNNs as those used in Fig 1C–E. (TIF) [file pcbi.1013083.s001.tif]

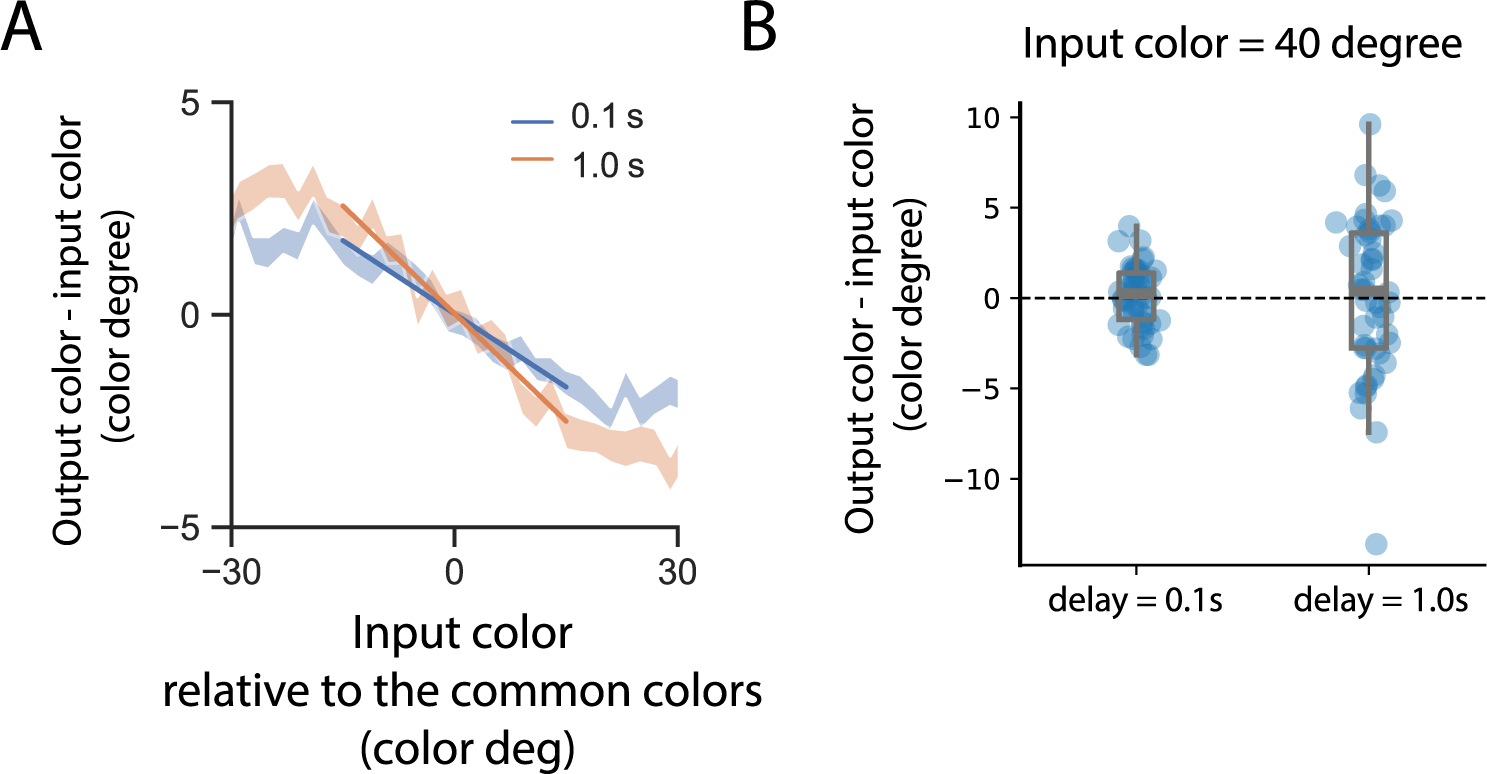

Supplement: S2 Fig — (A) Same as Fig 1E but generated with a different random seed. (B) For each Biased RNN, we conducted 1,000 trials with the input color fixed at a common color (40∘). We averaged the trials errors (output – input) across all trials resulting in a single averaged trial error of one RNN, represented by a single dot in this panel. We repeated the same procedure for 50 RNNs, and two delay lengths (0.1s and 1.0s). Boxes represent the first and third quartiles, with the central line indicating the median. Whiskers extend to the minimum and maximum values within 1.5 times the interquartile range. There’s no statistically significant difference between the group medians and zero (p>0.5, Wilcoxon signed-rank test, two-sided), in both delay of 0.1 s and 1.0 s. (TIF) [file pcbi.1013083.s002.tif]

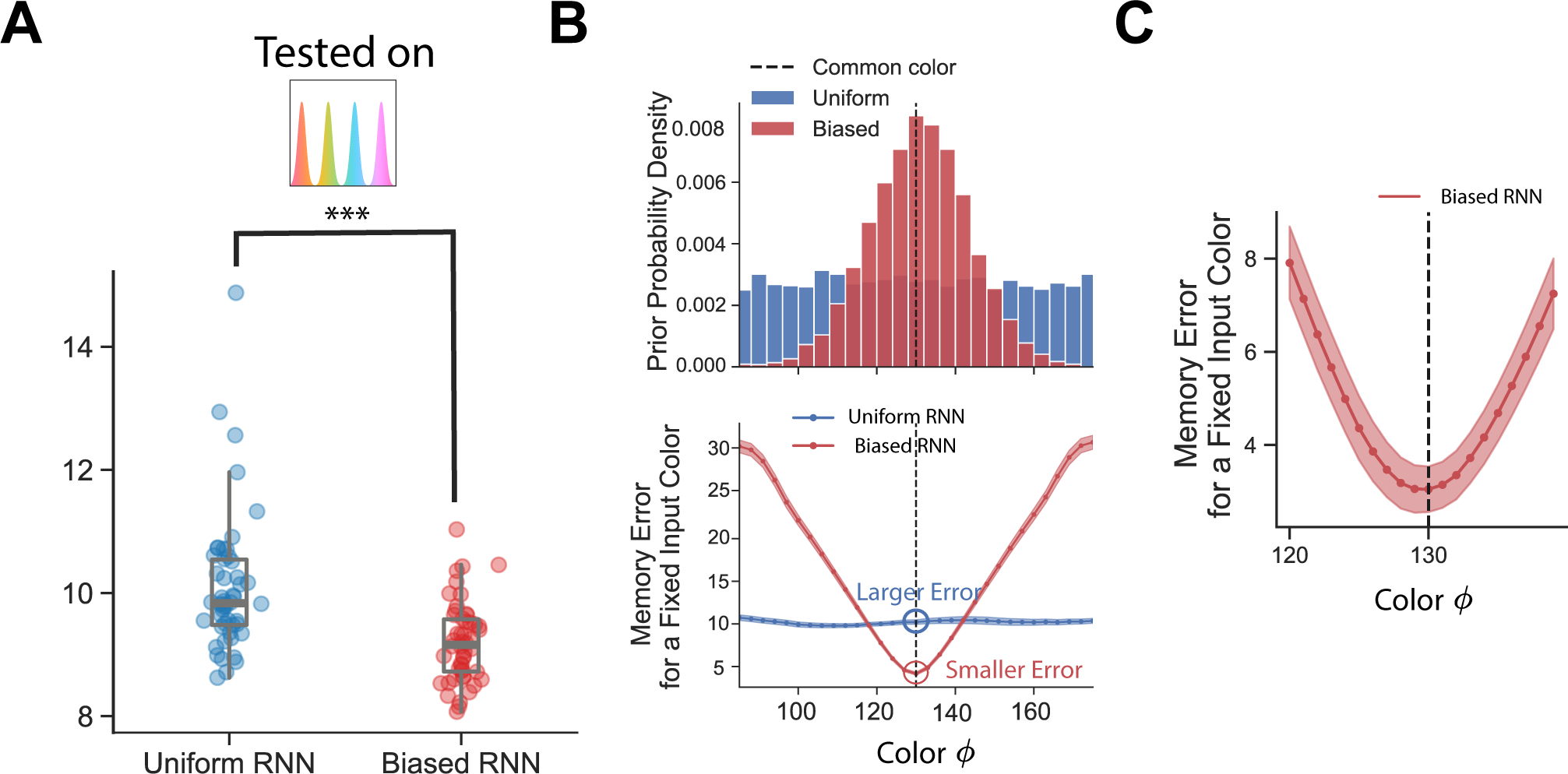

Supplement: S3 Fig — (A) Memory errors of trained Biased (σs=12.5∘) and Uniform RNNs in a biased environment (σs=12.5∘). Each RNN was run for 5,000 trials with input colors sampled from a biased environment (σs=12.5∘). Errors from outlier trials, defined as values exceeding 1.5 times the interquartile range above the third quartile or below the first quartile, were excluded. The memory error of each RNN was calculated as the RMSE across trials. Each dot represents one RNN, with 50 RNNs used for each type. (***: p<10−3, Wilcoxon rank-sum test, two tailed). (B) Top: The prior distribution of colors centered around a common color (130∘, black dashed line). Bottom: Memory error for various input colors. We ran 5,000 trials for each RNN (50 RNNs for each type) and each input color. Errors from outlier trials among the 5,000 trials were removed. The memory error of one RNN and one input color was computed as the square root of the average squared difference between the output and input colors across trials. The colored lines represent the mean memory errors across the 50 RNNs (after removing memory errors from outlier RNNs), with error bands indicating standard errors. (C) Same as the bottom of panel (B), but for Biased RNNs with all noise turned off. (TIF) [file pcbi.1013083.s003.tif]

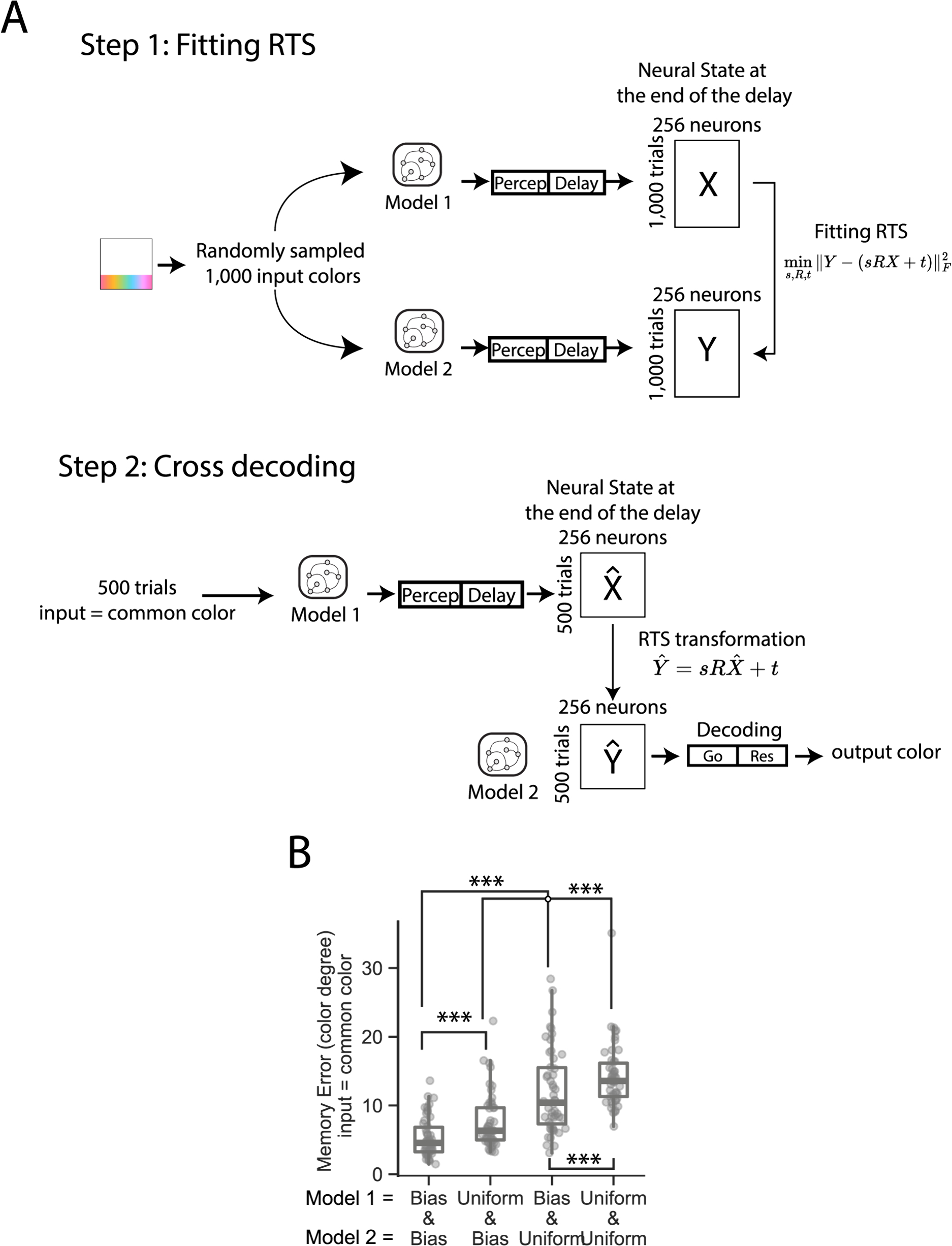

Supplement: S4 Fig — (A) Details of the method for performing cross-decoding on one pair of Model 1–Model 2 using rotation-translation-scaling (RTS) matching. In step one, 2,000 input colors were randomly sampled. Model 1 and Model 2 were run for 1,000 trials each, with neural states at the end of the delay collected and denoted as X and Y respectively. RTS matching finds the optimal scaling (s), rotation/reflection (R) and translation (t) to transform X by minimizing the Frobenius distance between the transformed X and Y. In step two, 500 trials were performed with input colors fixed to the common color. Model 1’s neural states at the end of delay (X^) were collected and transformed into Model 2’s representation (Y^). Model 2 ran through the post-delay epochs and finally generated output colors. (B) Same as Fig 2B in main text, but using RTS matching. (TIF) [file pcbi.1013083.s004.tif]

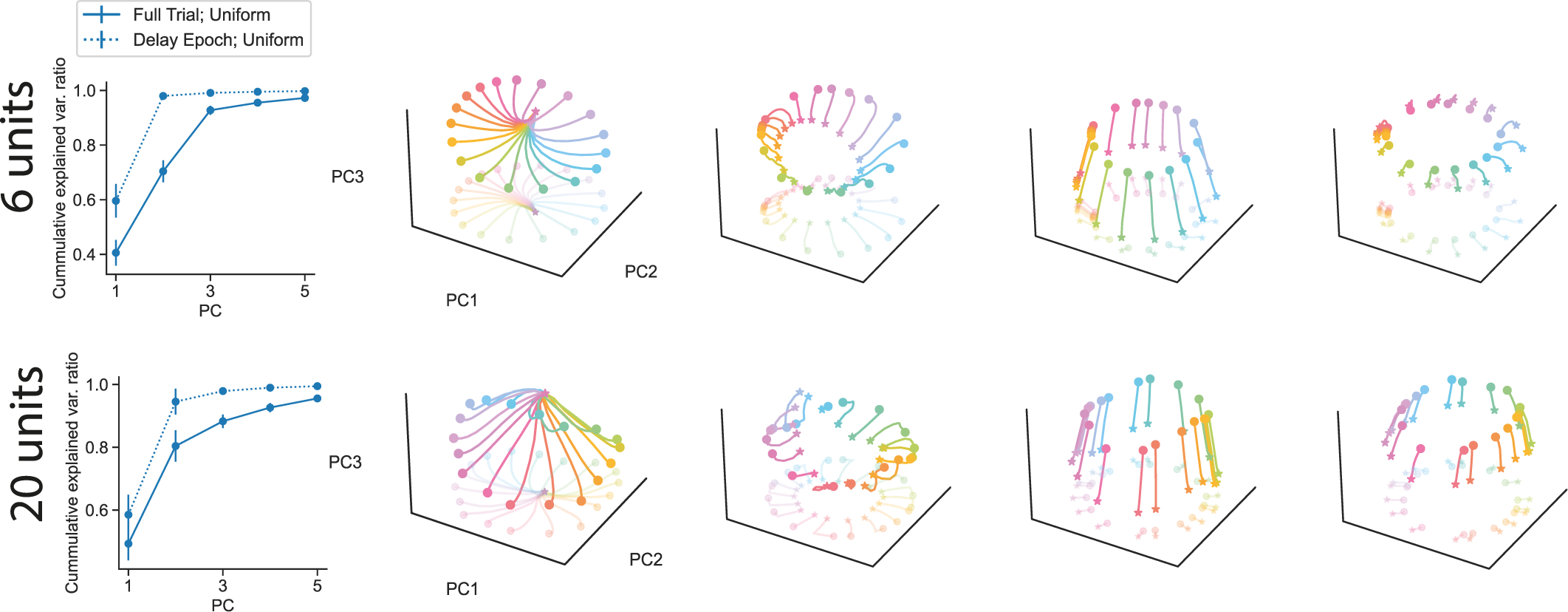

Supplement: S5 Fig — Figure illustrations are the same as those in Fig 3A and 3B. (TIF) [file pcbi.1013083.s005.tif]

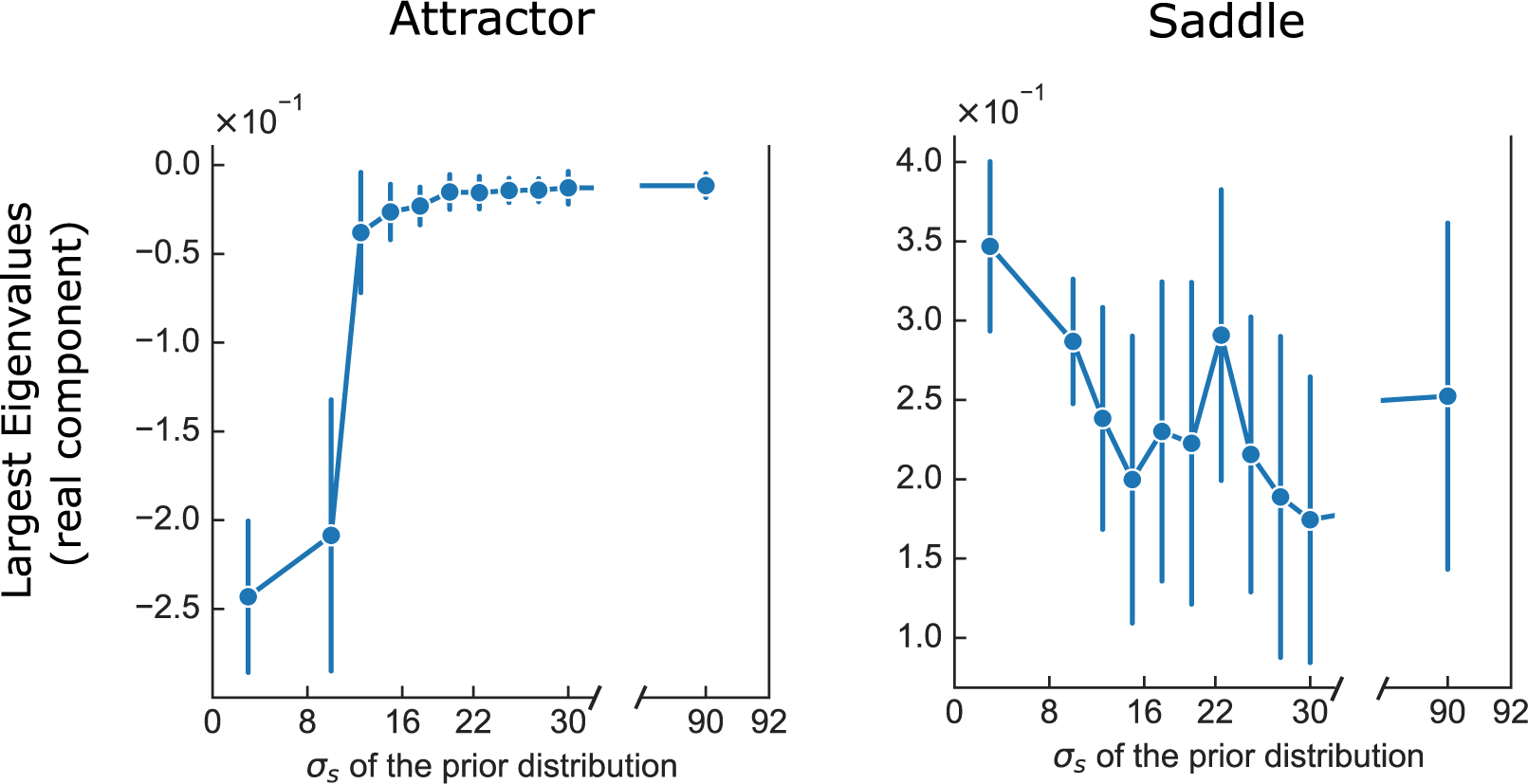

Supplement: S6 Fig — (Left) We trained 50 RNNs for each prior σs. For each trained RNN, we identified its attractors and calculated the largest eigenvalue for each attractor. These largest eigenvalues were then averaged to represent the “attracting strength” of the RNN. The dots and error bars represent the mean and standard deviation of the averaged largest eigenvalues across the 50 RNNs. (Right) Same as the left, but applied to the largest eigenvalues of the saddle points. These results indicate that RNNs trained with narrower priors (smaller σs) tend to form stronger fixed points, which may explain the neural clustering observed in Fig 3C and 3E. (TIF) [file pcbi.1013083.s006.tif]

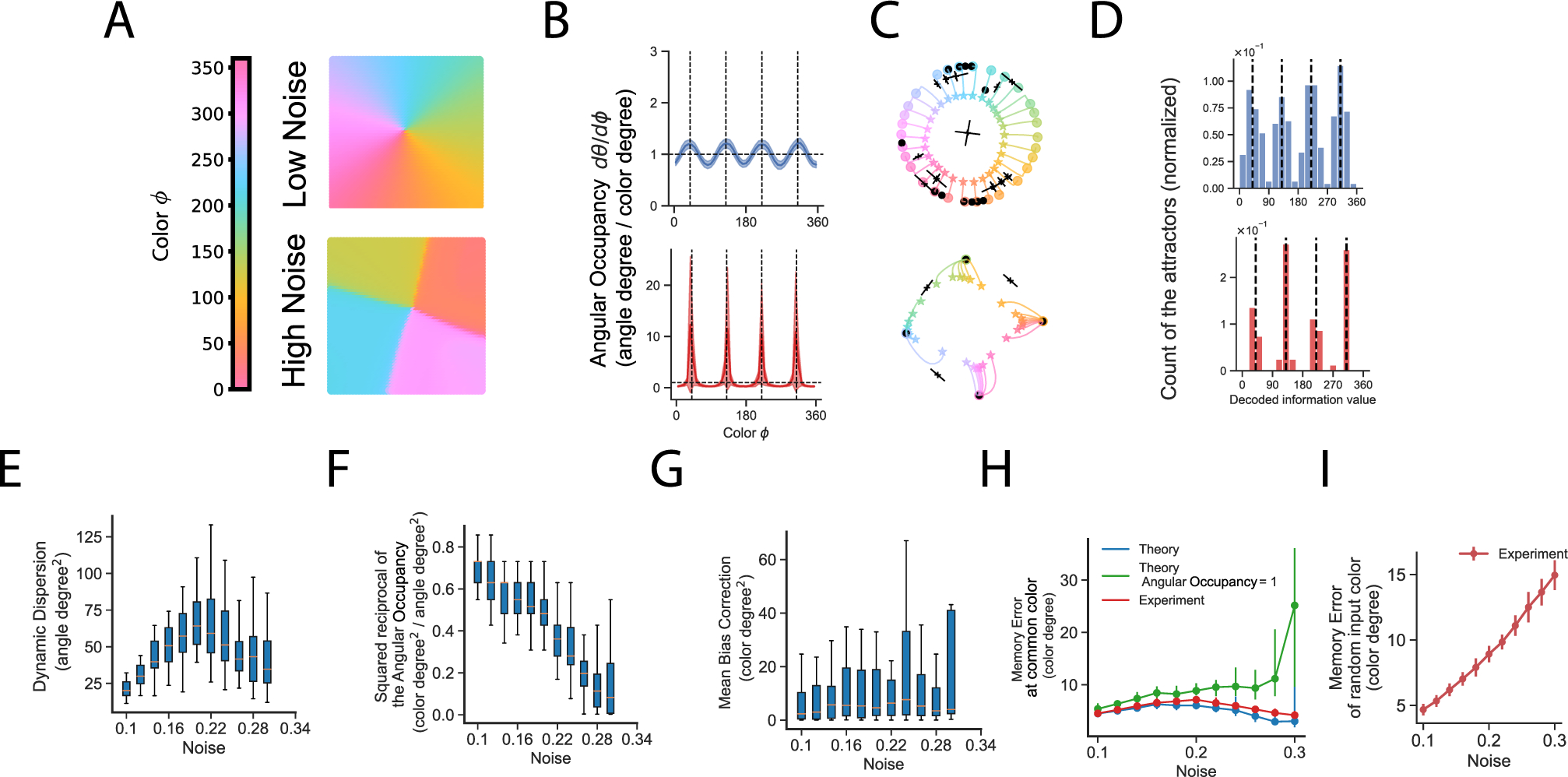

Supplement: S7 Fig — (A) The delay plane for two example RNNs trained on low-noise (noise strength σx=σrec=0.1) and high-noise (σx=σrec=0.3). Colors indicate the decoded colors (i.e., output colors) by continuing the neural states on the delay through the post-delay epochs. (B) Angular occupancy. Dashed vertical lines are common colors. Solid blue/red lines indicate the mean of 50 RNNs. The error bands indicate std. (C) Neural dynamics during delay. The color of each trajectory indicates the input color of a trial. Stars are the beginning of delay; dots are the ends. Black dots are attractors, black crosses are saddles with long bar indicating the positive eigenvalue directions. (D) Number of attractors concatenated from 50 RNNs (normalized) for different colors. Dashed lines: common colors. (E) Same as Fig 4B. Increasing noise initially increase dynamic dispersion (noise from 0.10 to 0.22, Spearman correlation = 0.69, p<10−3, two-tailed Spearman correlation test implemented by scipy.stats.spearmanr), but then decrease the dynamic dispersion (noise from 0.22 to 0.30, Spearman correlation = -0.33, p<10−3, two-tailed Spearman correlation test implemented by scipy.stats.spearmanr). The counter-intuitive of decreasing dynamic dispersion might be due to an over-powerful attracting mechanism to counteract the effect of noise. For example, consider an extreme scenario where the noise is very high, input color information is destroyed completely during the trials. In this case, the RNN’s optimal strategy would be to output common colors for any inputs, as common colors have high priors. Therefore, the RNN might develop very strong attracting mechanisms under high noise conditions such that every trial the neural states would be quickly attracted to attractors to output common colors. (F, G, H) Same as Fig 4C, 4D, and 4E, but varying noise level. Fifty RNNs were trained at each noise level. (I) Experimental error same as (H), but input colors were randomly sampled from an environmental prior σs= [file pcbi.1013083.s007.tif]

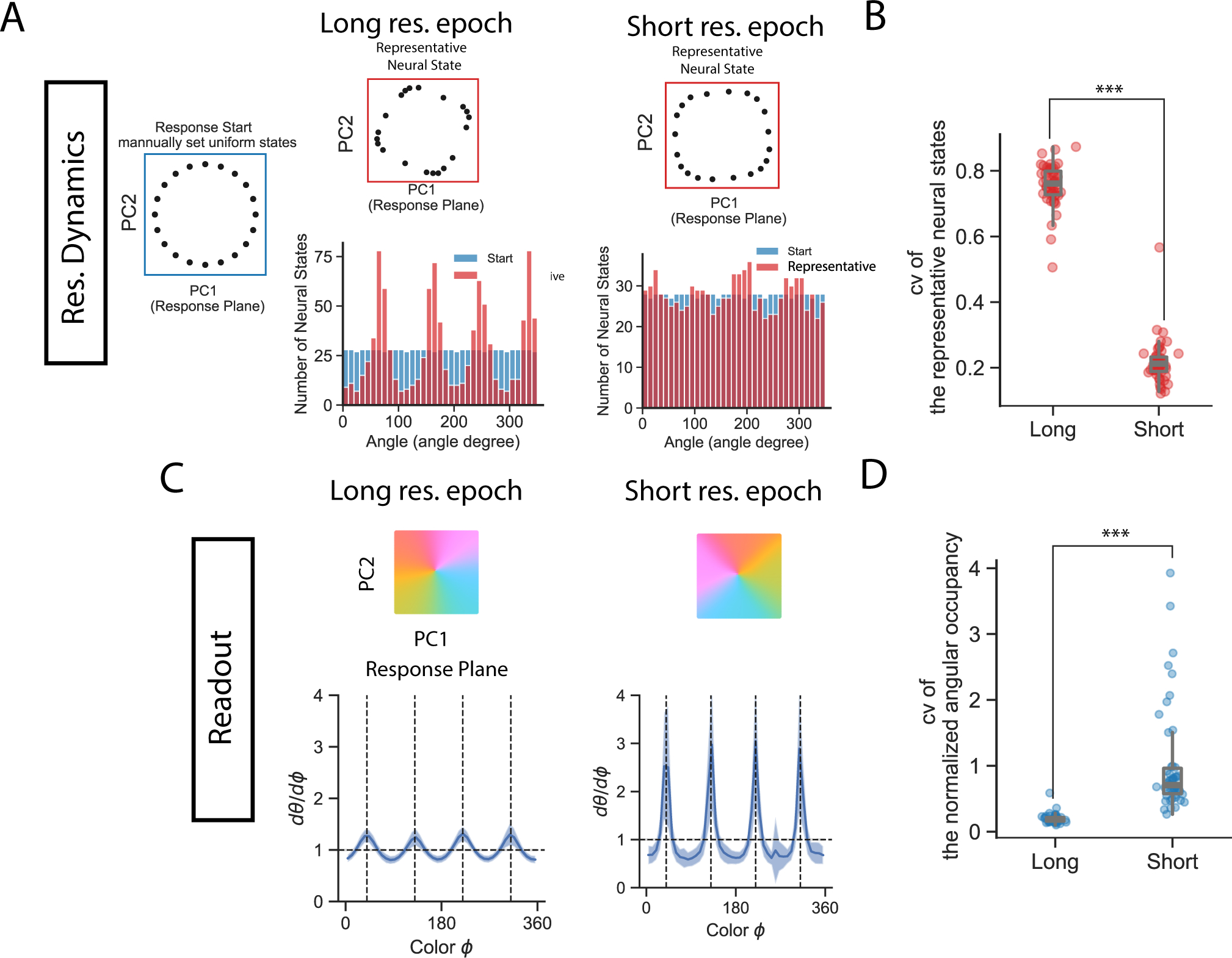

Supplement: S8 Fig — Training prior σs=3∘. The computational details are identical to those in Fig 5, except that the RNN models were replaced with those trained using long/short response (res.) epoch. (A) Long/short response epoch: an example RNN trained with a long (200 ms) or short (40 ms) response epoch length. Compared to the RNN trained with a long response epoch, RNN trained with a short response epoch shows smaller changes in neural states during the response epoch. (B) RNNs trained on long response epochs exhibit smaller bias as measured by the coefficient of variation (CVs) of representative neural states distributions (see Methods). Each dot represents one RNN’s representative neural state CV (red histogram in panel A). Boxes indicate the first, median and third quantiles across 50 RNNs. ***: p<10−3, Wilcoxon rank-sum test. (C) RNNs trained on short response epoch have more biased readout. The solid line represents the mean of 50 RNNs, and the shaded error band indicates the standard deviation. Dash lines: four common colors. Outlier RNNs were removed (outside of 1.5 IQR). (D) Same as panel (B), but measuring the CV of angular occupancies. (TIF) [file pcbi.1013083.s008.tif]

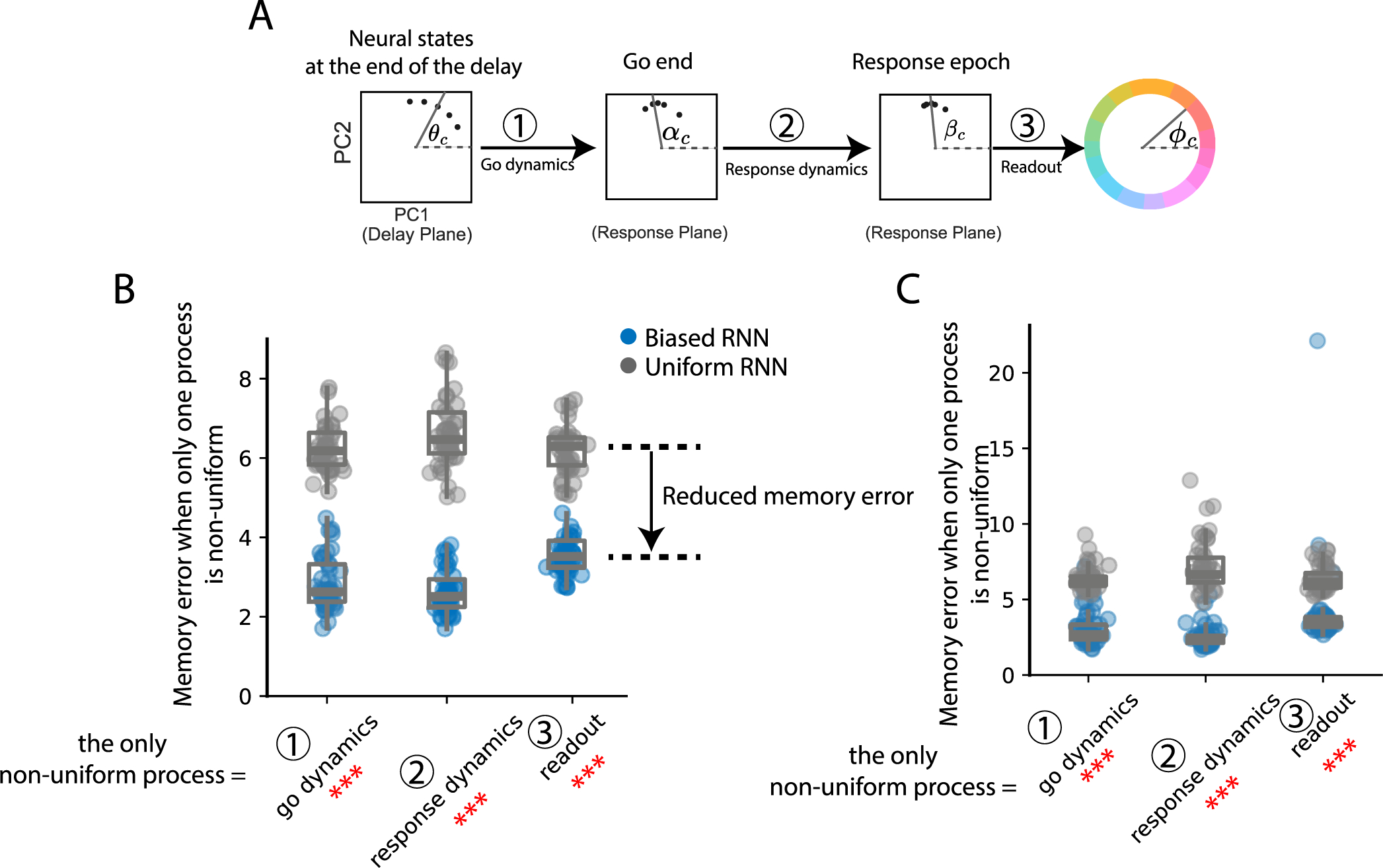

Supplement: S9 Fig — (A) The post-delay process was divided into three distinct processes: (1) neural states at the end of the delay evolve through go dynamics, (2) followed by response dynamics, and finally, (3) are read out into colors. Due to the low-dimensional structure of the trained RNN (Fig 3A), each process can be simplified as a non-linear transformation of angles. For instance, the effect of go dynamics can be represented as a transformation of angles on the delay plane into new angles on the response plane. The angles at each stage are denoted as θ (end of the delay = beginning of the post-delay epochs), α, β and φ (color, also ranges from 0∘ to 360∘), respectively. The angle corresponding to a common color is indicated with the subscript c. To isolate the effect of a single process on the memory of the common color, we allowed only that process to run in the RNN while manually keeping the other two processes uniform (see Methods). (B) The memory errors associated with each of the three processes in Biased RNNs (n = 50, training prior σs=3∘, the same as the example RNN demonstrated in Fig 5B, 5D, and 5F–H) are smaller than those observed in Uniform RNNs. This suggests that each of the three processes—go dynamics, response dynamics, and readout—plays a role in reducing memory error. ***: p<10−3, one-sided, Wilcoxon rank-sum test. Each dot is the result of one RNN. Outliers were omitted for visual clarity but were included in the statistical analysis. (C) Same as the panel B, but includes the visually omitted outlier RNNs in the panel B. (TIF) [file pcbi.1013083.s009.tif]
